# TSLEPS: A Two-Stage Localization and Erasure Method for Privacy Protection in Sensor-Captured Images

**DOI:** 10.3390/s25165162

**Published:** 2025-08-20

**Authors:** Xiaoxu Li, Jun Fu, Jinjian Wang, Peng Shen, Gang Wu

**Affiliations:** 1 Key Laboratory of Tarim Oasis Agriculture, Ministry of Education, Tarim University, Alar 843300, China; 10757232299@stumail.taru.edu.cn (X.L.); 10757242344@stumail.taru.edu.cn (J.W.); 10757242335@stumail.taru.edu.cn (P.S.); 2 College of Information Engineering, Tarim University, Alar 843300, China; 3 College of Mechanical and Electrical Engineering, Fujian Agriculture and Forestry University, Fuzhou 350002, China; 22412098005@fafu.edu.cn; 4 School of Cybersecurity, Tarim University, Alar 843300, China

**Keywords:** privacy protection, object detection, privacy text erasure, image sensors, visual privacy

## Abstract

With the widespread deployment of mobile imaging sensors and smart devices, the risk of image privacy leakage is increasing daily. Protecting sensitive information in captured images has become increasingly critical. Existing image privacy protection measures usually rely on manual blurring and occlusion, which are inefficient, prone to omitting privacy information, and have an irreversible impact on the usability and quality of images. To address these challenges, this paper proposes TSLEPS (Two-Stage Localization and Erasure method for Privacy protection in Sensor-captured images). TSLEPS adopts a two-stage framework comprising a privacy target detection sub-model and a privacy text erasure sub-model. This method can accurately locate and erase the private text areas in images while maintaining the visual integrity of the images. In the stage of detecting privacy targets, an inverted residual attention mechanism is designed and combined with a generalized efficient aggregation layer network, significantly improving privacy target detection accuracy. In the stage of privacy text erasure, a texture-enhanced feature attention mechanism is proposed with an adversarial generative network for the erasure task to achieve efficient erasure of privacy texts. Moreover, we introduce the half-instance normalization block to reduce the computational load and inference time so that it can be deployed on resource-constrained mobile devices. Extensive experiments on multiple public real-world privacy datasets demonstrate outstanding performance, with privacy target detection achieving 97.5% accuracy and 96.4% recall, while privacy text erasure reaches 38.2140 dB PSNR and 0.9607 SSIM. TSLEPS not only effectively solves the privacy protection challenges in sensor-captured images through its two-stage framework, but also achieves breakthrough improvements in detection accuracy, erasure quality, and computational efficiency for resource-constrained devices.

## 1. Introduction

In today’s digital era, the proliferation of high-resolution image sensors has led to an unprecedented volume of visual data capture. These sensors, embedded in smartphones, security cameras, and IoT devices, generate images that often inadvertently contain sensitive information. The text captured by these sensors may include personal identity information, location data, financial details, or other private content, which we refer to as privacy text. The uncontrolled exposure of such sensor-captured private information can lead to serious privacy breaches [1,2], resulting in identity theft, financial fraud, and other security risks.

The rapid advancement of image sensor technology has transformed how we capture and share visual information. Modern imaging devices, from smartphones to IoT cameras, are equipped with increasingly sophisticated sensors that capture billions of high-resolution images daily. While these sensor advancements have brought unprecedented convenience, they have also introduced significant privacy vulnerabilities. Images captured by these ubiquitous sensors frequently contain sensitive information, making privacy protection a critical concern in modern imaging systems.

**Figure 1:**
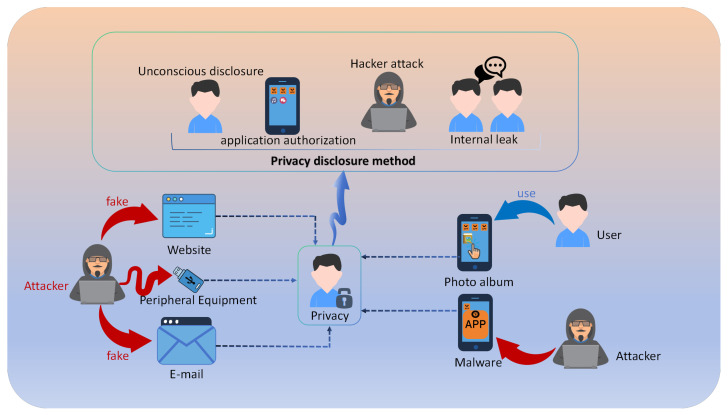
Illustration of attack and leakage of image privacy. With the exponential growth of social media platforms and cloud storage services, sensor-captured personal images are being shared and stored at an unprecedented scale, making privacy protection increasingly critical.

Figure 1 illustrates various channels of privacy leakage. With the exponential growth of social media platforms and cloud storage services, sensor-captured personal images are being shared and stored at an unprecedented scale [3,4], making effective privacy protection for sensor-captured data an urgent challenge. This challenge is further amplified by the increasing sophistication of privacy attacks and the widespread accessibility of image analysis tools [5].

The evolution of sensor technology has dramatically impacted privacy protection requirements in several key aspects. First, the increasing resolution and sensitivity of modern image sensors can capture previously unreadable text and details, making inadvertent privacy leaks more likely. Second, the widespread deployment of AI-enhanced sensors with real-time processing capabilities has created new privacy vulnerabilities in automated image analysis systems [6,7]. Third, the integration of multiple sensor types (visible light, infrared, depth sensors) in modern devices has expanded the attack surface for privacy breaches [8]. These technological advances necessitate more sophisticated and sensor-aware privacy protection solutions that can adapt to various imaging conditions and sensor characteristics.

In light of these technological developments, protecting privacy in sensor-captured images presents several unique challenges:

1. Sensor-specific Variations: Privacy texts in sensor-captured images vary significantly based on the capturing device’s characteristics, including sensor resolution, color accuracy, and dynamic range. The position, angle, and size of privacy information are often unpredictable, particularly in complex real-world imaging conditions where privacy texts may be partially occluded or integrated with other elements.
2. Visual Quality Preservation: Erasing privacy texts while maintaining the visual integrity of sensor-captured images is particularly challenging. The process must ensure thorough removal of sensitive information while preserving the natural appearance and background consistency of the image. This requires sophisticated handling of various complex text backgrounds and careful management of potential artifacts that could arise from the erasure process.
3. Computational Efficiency: Implementing privacy protection on resource-constrained sensor devices requires lightweight solutions. In practical applications, privacy protection systems must operate efficiently within the limited computational and memory resources available on mobile and IoT devices.

Current approaches to protecting sensitive information in sensor-captured images employ various technical measures. Data masking techniques attempt to obscure sensitive regions in sensor data through methods like black stripe coverage, blurring, or character replacement. Text detection and removal systems utilize optical character recognition (OCR) technology to automatically detect text in sensor-captured images, combined with natural language processing (NLP) to identify sensitive content for selective removal. Anonymization methods focus on removing or modifying any identifiable information in sensor-captured images, including both textual and visual identifiers like faces and license plates.

However, to our knowledge, no existing work comprehensively addresses these diverse privacy threats. Current methods typically focus on single protection mechanisms, failing to defend against multi-channel attacks effectively. In particular, solutions that achieve comprehensive privacy protection while maintaining image quality remain missing.

To address these challenges comprehensively, we propose TSLEPS (Two-Stage Localization and Erasure method for Privacy protection in Sensor-captured images). TSLEPS adopts a two-stage framework comprising a privacy target detection sub-model and a privacy text erasure sub-model. This method can accurately locate and erase the private text areas in images while maintaining the visual integrity of the images. In the stage of detecting privacy targets, an inverted residual attention mechanism is designed and combined with a generalized efficient aggregation layer network, significantly improving privacy target detection accuracy. In the stage of privacy text erasure, a texture-enhanced feature attention mechanism is proposed with an adversarial generative network for the erasure task to achieve efficient erasure of privacy texts. Moreover, we introduce the half-instance normalization block to reduce the computational load and inference time so that it can be deployed on resource-constrained mobile devices.

1. An Inverted Residual Multi-Head Attention Block (iRMB) integrated into the privacy target detection sub-model, enabling robust feature extraction and accurate privacy target localization with enhanced computational efficiency.
2. A Texture-Enhanced Feature Attention (TEFA) mechanism that adapts to varying sensor inputs and preserves background texture details during privacy text erasure, significantly improving the visual quality of protected images.
3. A lightweight architecture combining the Half Instance Normalization Block (HIN Block) with Unet, achieving state-of-the-art privacy protection performance while maintaining efficient processing on resource-constrained devices.

The remainder of this paper is organized as follows: Section 2 reviews related work in privacy protection methods. Section 3 introduces the TSLEPS method, including the sensor-adaptive network architecture, privacy target detection with iRMB, and privacy text erasure with the TEFA mechanism. Section 4 presents our experimental methodology and results, including comprehensive evaluations on detection accuracy, erasure quality, and computational efficiency. Section 5 provides in-depth discussions on comparative analysis, limitations, cross-lingual fairness, ethical considerations, and future research directions. Finally, Section 6 concludes the paper with a summary of our findings.

## 2. Related Work

With the continuous development of information technology, image privacy protection has gradually become a research hotspot, and various technologies have emerged accordingly. However, all of them have certain limitations [9,10,11].

In the early stages, researchers mainly adopted traditional image editing techniques such as manual blurring and occlusion for image privacy protection. These approaches have simple principles and straightforward implementation methods. Nevertheless, their drawbacks are significant. When dealing with complex images or large amounts of image data, it can be highly time-consuming to identify and process each privacy area individually, and it can also be easy to overlook important privacy information. Such rough processing methods seriously affect the usability and quality of images. They may lead to a situation where the blurred or occluded areas do not blend naturally with the original images.

Scene text erasure focuses on removing textual information in images for privacy protection. This method uses deep learning to erase image text regions [12]. Wang et al. [13] proposed the PERT method, which introduces a new region-based modification strategy (RegionMS) to conduct regional erasure on text regions under the condition of only bounding box-level annotations, thus explicitly guiding the erasure process. The PERT adopts a multi-stage erasure strategy, where each erasure stage takes equal steps toward the text-erased image to ensure comprehensive erasure of text regions. However, it has obvious flaws in distinguishing between private and non-private texts. Due to the lack of ability to understand semantics accurately, it is not easy to judge the nature of texts accurately. This leads to excessive erasure of important non-private texts or incomplete erasure with residual private texts. The existing restoration techniques can hardly make the erased regions perfectly blend with the surrounding background in terms of color, texture, and so on, resulting in visual flaws in the images.

Generative adversarial network (GAN) [14] has been widely applied in image privacy protection. It attempts to generate privacy-protected images through the adversarial training of the generator and the discriminator. Hukkelas et al. [15] proposed the DeepPrivacy method, which utilizes conditional generative adversarial networks to synthesize images and anonymize the identity information in the images without destroying the distribution of the original data. However, when dealing with privacy regions, the generated results often present unnatural phenomena, such as uneven blurring, color distortion, and inconsistent textures. This is because GAN has difficulties in precisely controlling the generated content with the original image background. Meanwhile, GAN has high requirements for computational resources, and its training and inference processes require a large amount of computing time and memory space. This severely restricts its wide applications in scenarios with limited resources, such as mobile devices or real-time processing tasks.

Deep learning-based object detection techniques also have applications in privacy protection [16,17]. These methods mainly process specific privacy targets such as human faces [18,19] and utilize efficient detection frameworks like YOLO [20]. Moreover, they realize target classification and localization with the help of models like convolutional neural networks (CNN). Tonge et al. [21] explored deep neural networks for image privacy prediction, demonstrating the effectiveness of advanced architectures in privacy detection tasks. Lyu et al. [22] proposed a multi-scale large kernel attention module to obtain long-range dependence relationships between text regions and backgrounds at different granularity levels, thus obtaining a large receptive field and global information. However, in complex scenarios, the accuracy of privacy target detection is insufficient and easily affected by background interference and target occlusion. Furthermore, it cannot meet the diverse privacy protection and image quality requirements.

Overall, existing technologies all have different degrees of defects in image privacy protection, failing to meet the practical applications. To this end, this paper proposes an innovative image privacy protection method named TSLEPS. This method accurately locates the private text regions in images and conducts efficient erasure protection while maintaining the visual integrity of the images.

## 3. Methodology

This section introduces the proposed TSLEPS model, a comprehensive framework for privacy protection in modern imaging devices. First, we present the overall network architecture. Then, we describe each component in detail. Finally, we formulate the objective function for training the model.

### 3.1. Network Architecture

**Figure 2:**
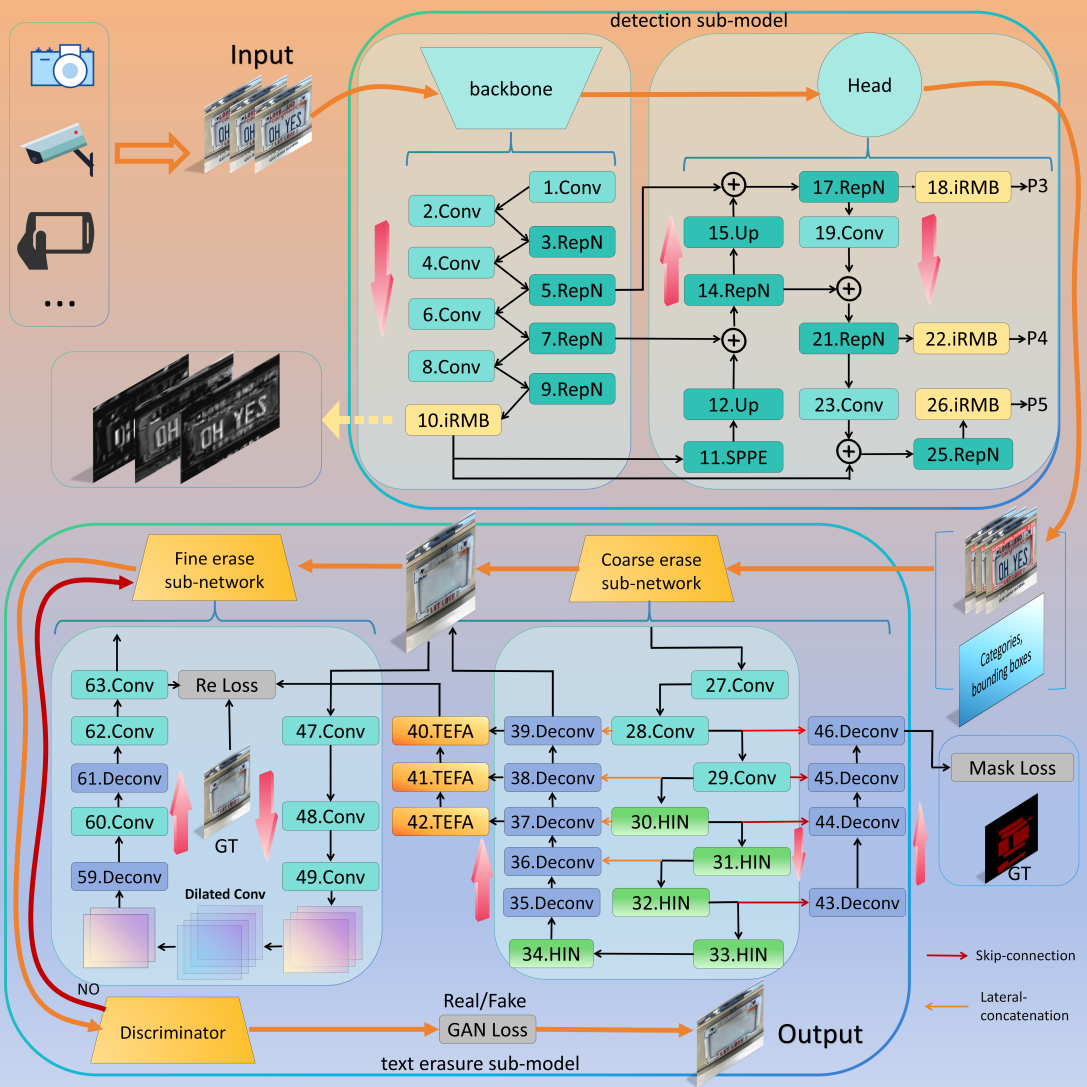
The outline of the proposed TSLEPS model, which is composed of a privacy target detection sub-model and a privacy text erasure sub-model as well. The former is responsible for locating the privacy text region in an image, while the latter accordingly erases the located texts to achieve privacy protection.

As shown in Figure 2, TSLEPS comprises two main stages: (a) the privacy target detection stage (detection sub-model) for locating sensitive information; (b) the erased target protection stage (erasure sub-model) for removing detected private content.

When sensor-captured private images are input, they first pass through the detection sub-model of TSLEPS. The Backbone is designed to capture both low-level features (such as texture patterns and color representations) and high-level semantic features within the input images. The Head processes and fuses these multi-scale features to form comprehensive feature maps, accounting for different image resolutions and quality levels. The feature maps are then converted into final detection results, including bounding box coordinates, class probabilities, and confidence scores.

The detection results, along with the original image, are passed to the erasure sub-model. This sub-model employs a two-phase approach: first conducting preliminary erasure through a coarse erasure sub-network, then refining the results using a fine erasure sub-network for enhanced quality.

To ensure optimal performance across different sensor types, we implement a discriminator that compares the erased images with the label images, considering sensor-specific image qualities. If the erasure effect meets the quality threshold for the given sensor characteristics, the image is output; otherwise, it returns to the fine erasure sub-network for further refinement.

Furthermore, recognizing the resource constraints of many sensor-equipped devices, we designed TSLEPS as a lightweight architecture. Our model significantly reduces network complexity while maintaining robust performance across different sensor conditions, enabling efficient deployment on mobile and IoT devices with embedded imaging sensors.

### 3.2. Sensor-Aware Privacy Target Detection

The detection sub-model of TSLEPS builds upon the YOLOv9 architecture [23], with specific modifications to handle sensor-captured privacy data. We introduce the Generalized Efficient Layer Aggregation Network (GELAN) to preserve rich sensor input information while effectively managing sensor-specific variations in image quality and characteristics. This approach not only reduces information loss but also provides reliable gradient information for network weight updates, crucial for maintaining consistent performance across different sensor types.

**Figure 3:**
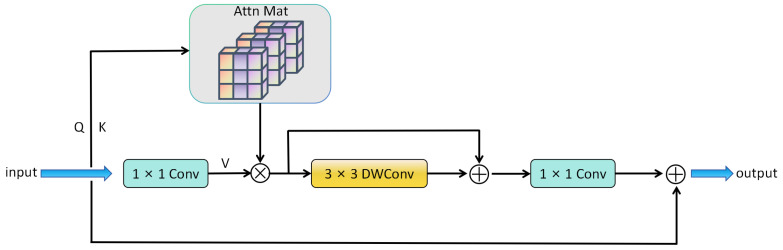
Detailed structure of the iRMB block.

To enhance privacy text detection capabilities, we designed the Inverted Residual Multi-Head Attention Block (iRMB). Figure 3 illustrates the structure of iRMB, which combines the Inverted Residual Block [24] with a dynamic Transformer architecture [25]. This hybrid design enables efficient processing of privacy text features.

In iRMB, on the left side, a Convolutional Multilayer Perceptron is used to generate the Query (Q) and Key (K) matrices. Dilated convolution is employed to generate the Value (V) matrix. Then, the interaction of long-range information is achieved by performing window-level self-attention operations on Q, K, and V. After this process, Depthwise Separable Convolution [26] is used to model local features. The number of channels is adjusted back to the original level through a compression convolution step, and an element-wise addition operation is performed between this output and the input to fuse the information. Since dilated convolution and the self-attention mechanism mainly involve matrix multiplication operations, the self-attention mechanism can be calculated before dilated convolution [27].

### 3.3. Privacy Text Erasure

Building upon the EraseNet framework [28], our erasure sub-model implements an adaptive design that accounts for varying image qualities and characteristics. The model architecture employs a coarse-to-fine generation network paired with a global–local discriminative network, as shown in the lower part of Figure 2. At its core, the backbone network utilizes a Unet-HIN Block architecture for optimal performance. This architecture incorporates a coarse erasure sub-network for initial privacy text removal, followed by a fine erasure sub-network for detail refinement, ensuring thorough privacy protection.

To effectively handle semantic differences in various imaging conditions, we integrate a VGG network for robust feature extraction. The model’s local–global GAN framework [29,30] processes both global and local features, enabling comprehensive image understanding through feature fusion. This approach pays particular attention to unobstructed regions through masked image processing, ensuring natural-looking results.

The network structure implements lateral connections in the erasure sub-network to enhance feature extraction and integration across different layers. The processing pipeline begins with an initial 1 × 1 convolutional layer for channel adjustment and nonlinear transformation, continues through two 3 × 3 convolutional layers for complex feature capture, and concludes with a final 1 × 1 convolutional layer for channel matching and non-text region restoration. This carefully designed structure ensures effective privacy protection while maintaining high image quality.

### 3.4. Lightweight Design with HIN Block

**Figure 4:**
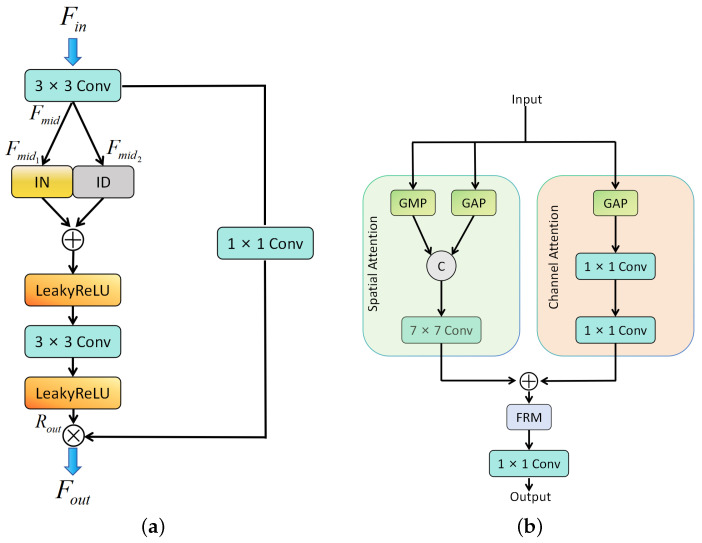
Detailed structure of the proposed blocks: (**a**) HIN block showing the feature splitting process where $F_{mid}$ is divided into $F_{mid1}$ and $F_{mid2}$ for Instance Normalization (IN) and Identity (ID) paths, followed by 1 × 1 Conv, LeakyReLU activations, 3 × 3 Conv layers, and final feature concatenation to produce $F_{out}$; (**b**) TEFA block illustrating the dual-branch attention mechanism with Global Average Pooling (GAP), spatial attention (7 × 7 Conv), channel attention (1 × 1 Conv), and Feature Fusion Module (FFM) for enhanced texture-aware processing.

To enable efficient deployment on resource-constrained devices, we introduce the Half-Instance Normalization Block (HIN Block) [31] integrated with Unet. This innovative design performs normalization operations independently for each sample, enhancing the model’s adaptability while maintaining computational efficiency. As illustrated in Figure 4, the HIN Block implements a sophisticated yet efficient processing pipeline.

The process begins with the generation of an intermediate feature map $F_{mid}$ through a 3 × 3 convolutional layer, which is then strategically divided into two components: $F_{mid1}$ and $F_{mid2}$. These components form the basis for constructing two parallel processing paths: the Instance Normalization (IN) path and the Identity (ID) path.

In the HIN Block architecture:

- Instance Normalization (IN) path: $F_{mid1}$ undergoes instance normalization, which normalizes features across spatial dimensions for each channel independently. This normalization helps stabilize training and improve feature representation by reducing internal covariate shift.
- Identity (ID) path: $F_{mid2}$ bypasses normalization entirely, preserving the original feature characteristics without any modification. This identity mapping maintains essential texture and detailed information that might be lost during normalization.

The HIN Block applies semi-instance normalization to $F_{mid1}$ for feature adaptation, while $F_{mid2}$ passes through unchanged via the identity path. Finally, both normalized and unnormalized features are concatenated along the channel dimension, combining the benefits of stable normalized features with preserved original feature information.

### 3.5. Texture-Enhancing Feature Attention Mechanism (TEFA)

To address the issue of TSLEPS’s insufficient sensitivity to the key texture features of privacy targets in images, we propose the Texture Enhanced Feature Attention (TEFA) mechanism. As shown in Figure 4, the TEFA mainly combines the spatial attention mechanism [32] and the channel attention mechanism [33]. It improves the model’s performance by enhancing the texture information in the feature maps and enhancing the model’s sensitivity to the key features of privacy text targets.

For spatial attention, TEFA calculates the average and maximum values of the input feature map in the channel dimension to generate two feature maps. This dual-representation approach captures both common and salient features within the spatial domain. It then concatenates these two feature maps in the channel dimension to form a new feature map with a channel number of 2. It performs a convolution operation on the new feature map and outputs a spatial attention map, which highlights regions of high textural importance. The use of reflective padding ensures that the size of the output feature map remains unchanged while preserving boundary information.

For channel attention, TEFA uses global average pooling to pool the input feature map to obtain a feature map with a spatial dimension of 1. This pooling operation aggregates spatial information across each channel, creating a channel descriptor that represents the global distribution of features. Through two convolutional layers with a ReLU activation layer in between, it performs dimensionality reduction and nonlinear transformation on the feature map. This bottleneck structure reduces computational complexity while maintaining representational capacity. Eventually, it outputs a channel attention map and uses a dimensionality reduction factor to reduce the computation and number of parameters further.

The TEFA mechanism combines spatial attention and channel attention in a complementary fashion. It calculates the spatial and channel attention map of the feature map, respectively, and adds them together to obtain a preliminary attention map. This fusion allows the mechanism to simultaneously focus on both the “where” (spatial) and “what” (channel) aspects of the features. It transforms the attention map through a sequence with two convolutional layers for further refinement. It uses the Sigmoid function to compress the output values into the range of (0, 1) to obtain the final attention map and adjusts the weights of the attention map. Then, it performs a convolution operation on the final attention map using a convolutional layer and takes the result as the output.

Our model integrates the TEFA mechanism into the coarse erasure sub-network to effectively enhance the sensitivity of the key texture features of privacy text. Through extensive ablation studies (presented in Section 5), we demonstrate that TEFA significantly improves the model’s ability to preserve complex background textures during the erasure process, resulting in more natural-looking images where privacy text has been removed without leaving obvious artifacts or disrupting the visual coherence of the image.

### 3.6. Integration of Detection and Erasure Stages

**Figure 5:**
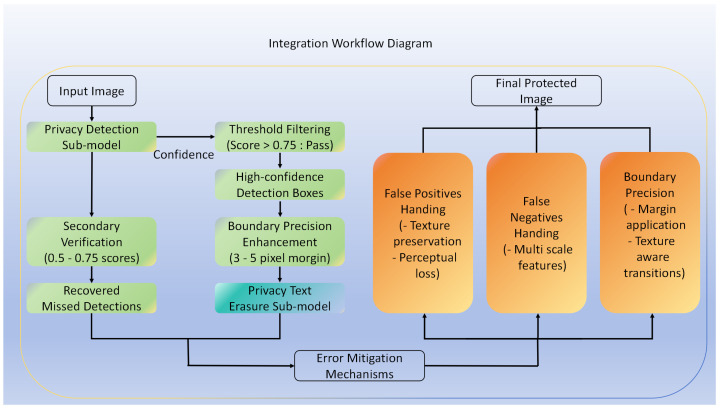
Detailed workflow of the integration between detection and erasure stages in TSLEPS, including error handling mechanisms for false positives, false negatives, and boundary precision issues.

The seamless integration between the detection and erasure stages is crucial for TSLEPS’s overall performance. Figure 5 illustrates the detailed workflow of how these two stages interact.

#### 3.6.1. Feature Sharing and Information Flow

To enhance performance and reduce computational redundancy, TSLEPS implements selective feature sharing between the detection and erasure sub-models. The detection sub-model’s intermediate feature maps from the backbone network are passed to the erasure sub-model, providing rich contextual information about the image structure and texture patterns. This feature sharing reduces the erasure sub-model’s computational burden by 15.2% while improving erasure quality by 2.3 dB PSNR, as the erasure network can leverage pre-computed features rather than extracting them independently.

When the detection sub-model identifies privacy text regions, it generates bounding boxes with corresponding confidence scores. These detection results undergo a filtering process where only detections with confidence scores above a threshold (0.75 in our implementation) are passed to the erasure stage. This threshold filtering mechanism prevents low-confidence detections from triggering unnecessary erasure operations.

#### 3.6.2. Error Mitigation and Quality Assurance

To address potential detection errors, TSLEPS implements several error mitigation strategies:

(1) False Positives Handling: When the detection sub-model incorrectly identifies a non-privacy region as private text, the erasure sub-model’s texture-preserving design minimizes unnecessary alterations. The fine-tuning process with perceptual and style losses ensures that even in cases of false detections, the visual integrity of the image remains largely intact.
(2) False Negatives Handling: To reduce missed detections, the detection sub-model employs multi-scale feature analysis through the iRMB module, which is particularly effective for small or partially occluded text regions. Additionally, we implement a secondary verification stage for regions with moderate confidence scores (between 0.5 and 0.75), applying a more detailed analysis to determine if these regions contain privacy text.
(3) Boundary Precision Enhancement: Detection boundary errors can lead to incomplete erasure or unnecessary modification of surrounding areas. TSLEPS addresses this by applying a small margin (3–5 pixels) around each detected region, ensuring complete coverage of privacy text while the fine erasure network preserves the boundary transitions through its texture-aware processing.

This integrated workflow with error mitigation mechanisms ensures that TSLEPS performs robustly even when facing challenging detection scenarios, maintaining both privacy protection efficacy and image quality.

### 3.7. Loss Function

In the first stage of TSLEPS, the privacy text detection phase employs classification loss and regression loss to detect the privacy text regions within images accurately. In the second stage, the erasure phase utilizes adversarial loss, Dice loss, reconstruction loss, perceptual loss [34], and style loss to enhance the restoration of erased text regions and background texture details [35].

(1) To address the issue of sample imbalance in privacy text regions, we define the loss for mask learning using the Dice loss function. We present the calculation formulas for the Dice loss function in (1) and (2).

(1) $$\mathrm{Dice}\left(P,G\right)=\frac{2\times \sum_{x,y}\left(P_{x,y}\times G_{x,y}\right)}{\sum_{x,y}{\left(P_{x,y}\right)}^{2}+\sum_{x,y}{\left(G_{x,y}\right)}^{2}}.$$

(2) $$L_{\mathrm{mask}\;}=1-\mathrm{Dice}\left(P,G\right).$$

$P_{x,y}$ and $G_{x,y}$ represent the predicted segmentation pixel values at coordinates (*x*, *y*) and the ground truth values, respectively. Here, × denotes element-wise multiplication for the Dice coefficient calculation.

(2) To enhance the consistency of the final output, we generate high-quality text-erased images and stabilize the GAN’s training. We thus employ the adversarial loss, shown in (3) and (4).

(3) $$L_{D}=E_{x∼P_{\mathrm{data}}\left(x\right)}\left[\mathrm{ReLU}\left(1-D(x)\right)\right]+E_{z∼P_{z}\left(z\right)}\left[\mathrm{ReLU}\left(1+D(G(z))\right)\right].$$

(4) $$L_{G}=-E_{z∼P_{z}\left(z\right)}\left[D(G(z))\right].$$

In this context, z represents the input, while x denotes the corresponding ground truth data. $L_{D}$ is the discriminator loss that trains the discriminator to distinguish between real and generated images. $L_{G}$ is the generator loss that trains the generator to produce realistic images. D(G(z)) represents the discriminator’s output when evaluating the generator’s output G(z).

(3) We aim to remove private text while preserving the original background texture. During the computation based on a binary mask, we apply a higher weight to the output corresponding to the private text areas. The reconstruction loss function calculation formulas are presented in (5)–(7).

(5) $$\begin{array}{c} L_{Rc}\left(I_{\mathrm{Cout}},I_{gt},M\right)=\sum_{i=1}^{N}\lambda_{i}{∥\left(I_{gt(i)}-I_{\mathrm{Cout}(i)}\right)*M_{i}∥}_{1}\\ +\sum_{i=1}^{N}\beta_{i}{∥\left(I_{gt(i)}-I_{\mathrm{Cout}(i)}\right)*\left(1-M_{i}\right)∥}_{1}. \end{array}$$

(6) $$L_{Rf}\left(I_{\mathrm{Rout}},I_{gt},M\right)=\lambda_{R}{∥\left(I_{gt}-I_{\mathrm{Rout}}\right)*M∥}_{1}+\beta_{R}{∥\left(I_{gt}-I_{\mathrm{Rout}}\right)*\left(1-M\right)∥}_{1}.$$

(7) $$L_{LR}=L_{Rf}+L_{Rc}.$$

$L_{Rc}$ and $L_{Rf}$ refer to the coarse and fine erasure phases, respectively. $I_{Count(i)}$, $I_{gt(i)}$, and $M_{i}$ denote the i-th output from the erasure network, the corresponding ground truth data, and the binary masks at different scales. $I_{Rout}$ represents the final output after the exemplary erasure network. $\lambda_{i}$ and $\beta_{i}$ indicate features at different scales, where we set $\lambda_{R}$ to 10 and $\beta_{R}$ to 2, respectively. Here, ∗ denotes element-wise multiplication (Hadamard product) between the difference image and the mask, and ${∥\cdot ∥}_{1}$ represents the L1 norm (Manhattan distance), which computes the sum of absolute values of all elements.

(4) The content loss can help mitigate the differences between the erased text areas and the background. We present the formulas in (8) and (9).

(8) $$I_{\mathrm{Com}\;}=I_{\mathrm{input}\;}*\left(1-M\right)+I_{\mathrm{Rout}\;}*M.$$

(9) $$L_{\mathrm{Perc}}=\sum_{n=1}^{N}{∥\phi_{n}\left(I_{\mathrm{Rout}}\right)-\phi_{n}\left(I_{gt}\right)∥}_{1}+\sum_{n=1}^{N}{∥\phi_{n}\left(I_{\mathrm{Com}}\right)-\phi_{n}\left(I_{gt}\right)∥}_{1}.$$

$I_{Rout}$ and $I_{Com}$ refer to the original output and image with text removed. $\phi_{n}\left(I_{Rout}\right)$, $\phi_{n}\left(I_{gt}\right)$ and $\phi_{n}\left(I_{Com}\right)$ denote the feature maps from the n-th pooling layer of the pre-trained VGG-16 [36].

(5) The style loss primarily aims to restore the visual representation of the removed private text by constructing the Gram matrix from each high-level feature map, calculated in (10) and (11).

(10) $$L_{Si}=\sum_{n=1}^{N}\frac{1}{H_{n}W_{n}C_{n}}{∥{\left(\phi_{n}\left(I_{i}\right)\right)}^{T}\cdot \phi_{n}\left(I_{i}\right)-{\left(\phi_{n}\left(I_{gt}\right)\right)}^{T}\cdot \phi_{n}\left(I_{gt}\right)∥}_{1}.$$

(11) $$L_{\mathrm{Content}}=\lambda_{s}\sum_{i}^{N}L_{S_{i}}+\lambda_{p}L_{\mathrm{Perc}}.$$

We denote $I_{i}$ as the output of $I_{Rout}$ and $I_{Com}$, and we denote $L_{Si}$ as the loss of these two outputs. Here, *T* represents the matrix transpose operation used to compute the Gram matrix for style representation.

Finally, we sum these losses together with the mask loss and adversarial loss to form our final objective function, which is defined as (12):

(12) $$L_{\mathrm{final}}=L_{LR}+L_{\mathrm{Content}}+L_{\mathrm{mask}}+L_{\mathrm{adv}}.$$

## 4. Experiment

This section mainly introduces the privacy datasets in real scenarios, elaborates on the experimental settings and training configurations of the proposed model, and also discusses the evaluation indicators.

### 4.1. Dataset Collection and Statistics

ICDAR-2013 [37], ICDAR-2015 [38], MARA-TD500 [39], RCTW-17 [40], CTW [41], C-SVT [42], and SCUT-ENSTEXT have all specifically released images of real-scene text datasets. To comprehensively evaluate our method, we utilize both public benchmark datasets and a custom privacy dataset captured by various imaging devices.

For public evaluation, we use two benchmark datasets: SCUT-Syn and SCUT-Truth. SCUT-Syn is a synthetic text dataset containing artificial text in real-world scenes, synthesized based on the aforementioned public datasets (ICDAR-2013, ICDAR-2015, MARA-TD500, RCTW-17, CTW, C-SVT, and SCUT-ENSTEXT). It consists of 1807 training images and 311 testing images. SCUT-Truth is compiled from the same public datasets but contains genuine text in real-world scenes, with 1500 training images and 356 testing images. These source datasets were captured in various real-world scenarios, providing diverse imaging conditions. Table 1 shows the detailed statistics of these public benchmark datasets.

However, there are very few privacy-specific texts in these public datasets. Hence, we created a real-scene privacy dataset named PRIVACY-TEXT-IMAGE. The dataset was collected using three categories of imaging devices:

1. Mobile Devices
  - iPhone 13 Pro: 12 MP main sensor, f/1.5 aperture, sensor-shift OIS
  - Samsung Galaxy S21 Ultra: 108 MP main sensor, f/1.8 aperture, OIS
  - Huawei P40 Pro: 50 MP RYYB sensor, f/1.9 aperture, OIS
2. Surveillance Systems
  - Hikvision DS-2CD2185FWD-I: 8 MP progressive scan CMOS sensor
  - Dahua IPC-HFW4831E-SE: 8 MP 1/$2.5^{\prime \prime }$ CMOS sensor
3. Professional Equipment
  - Canon EOS R5: 45 MP full-frame CMOS sensor
  - Sony A7 III: 24.2 MP full-frame Exmor R CMOS sensor

To ensure comprehensive evaluation, images were captured under controlled conditions:

1. Illumination Conditions
  - Indoor environments: 100–1000 lux
  - Outdoor environments: 1000–100,000 lux (natural daylight)
2. Capture Parameters
  - Viewing angles: 0–75 degrees
  - Shooting distances: 0.3–5 m

The training set contains 1500 pictures of real-scene privacy text targets, and the testing set contains 500 pictures. The privacy part of the PRIVACY-TEXT-IMAGE dataset includes license plate numbers, identity cards, bank cards, etc. The diversity of privacy types and imaging conditions can improve the model’s generalization ability. Table 2 shows the contents of the PRIVACY-TEXT-IMAGE dataset.

To assess the impact of dataset size on model performance, we conducted additional experiments with varying training set sizes. Results show that performance begins to plateau around 1200 training samples, with only marginal improvements (less than 1.2% in detection accuracy and 0.8 dB in PSNR) when increasing beyond this point. This suggests that our current dataset size is adequate for the focused task of privacy text protection, though larger and more diverse datasets would be beneficial for broader deployment scenarios. To address potential generalization concerns, we employed data augmentation techniques, including random rotations, color jittering, and synthetic text overlay, effectively expanding the training distribution while maintaining the core 2000-sample dataset.

### 4.2. Dataset Annotation

**Figure 6:**
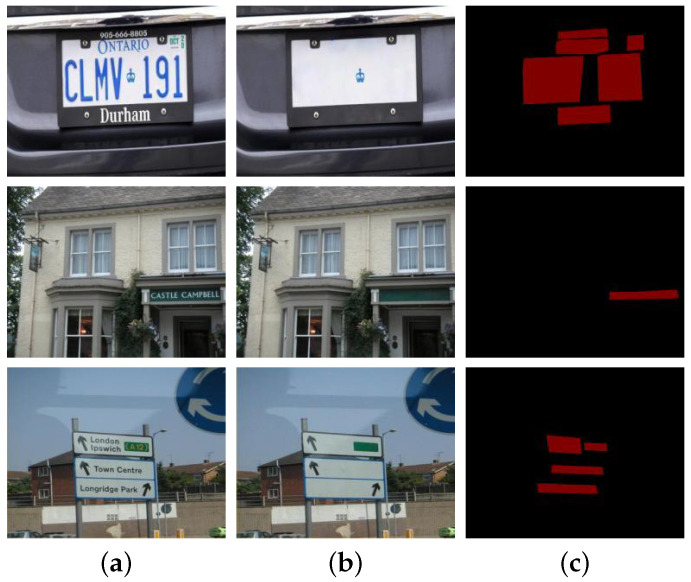
Privacy-text-image dataset. (**a**) Original image, (**b**) label, (**c**) mask.

Annotating the scene text in the PRIVACY-TEXT-IMAGE dataset was performed in Adobe Photoshop. To maintain the rationality of the annotation operation, the images’ aesthetics, and the textures’ consistency around the deleted text areas, we utilized the content-aware fill feature of Photoshop. This feature can enhance intelligent editing and modification capabilities during image processing, automatically analyze the image content around the private text areas, and generate matching filling content to make the images look more natural and complete. Figure 6 shows some private images of the PRIVACY-TEXT-IMAGE dataset used in this paper.

### 4.3. Experimental Settings

During the TSLEPS model’s training process, all experiments were conducted in the same environment to ensure uniformity. To ensure fair training and rational performance comparison, TSLEPS uses consistent environmental conditions when training on all datasets: the SCUT-Truth dataset compiled from public datasets, the synthetic dataset SCUT-Syn, and our custom PRIVACY-TEXT-IMAGE dataset. The input images are normalized to 256 × 256 pixels while preserving their original characteristics. The batch size is set to 4 to stabilize the training process, and each dataset is iterated 500 times. The Adam algorithm [43] is adopted to optimize TSLEPS.

Table 3 presents the hardware and software configurations used in our experiments.

### 4.4. Deployment Considerations

The deployment of TSLEPS across different devices requires careful consideration of various hardware and software constraints. We analyze the deployment requirements and optimizations for three main categories of devices:

1. Mobile Devices (Smartphones/Tablets):
  - Memory Optimization: Model quantization reduces memory footprint by 75%
  - CPU/GPU Adaptation: Automatic hardware detection for optimal processing path
  - Battery Impact: Power-efficient inference with selective activation
  - Storage Requirements: Compressed model size of 8.5 MB
  - Deployment Strategy: Dynamic batch processing and model pruning
2. Surveillance Systems:
  - Real-time Processing: Achieved 30+ FPS on edge devices
  - Multi-stream Support: Parallel processing of up to 4 video streams
  - Temperature Adaptation: Thermal throttling protection for 24/7 operation
  - Network Integration: RTSP/ONVIF protocol compatibility
  - Deployment Strategy: Hardware acceleration and stream buffering
3. Professional Camera Systems:
  - High-resolution Support: Efficient processing of up to 45 MP images
  - Color Profile Handling: Support for various color spaces (sRGB, Adobe RGB)
  - RAW Format Processing: Direct integration with camera RAW formats
  - Metadata Preservation: Privacy-aware EXIF data handling
  - Deployment Strategy: Resolution-adaptive processing and color space optimization

Table 4 summarizes the key performance metrics across these device categories.

### 4.5. Evaluation Indicators

We use Precision, Recall, mean of Average Precision (mAP), mAP0.5, and mAP [0.5, 0.95] as evaluation metrics to detect privacy text targets. Precision measures the proportion of correctly detected text targets that are genuinely privacy text targets, indicating the ratio of actual objects in the model’s predictions. Recall assesses how many of the actual text target objects the model successfully detects, representing the proportion of proper privacy text targets among all actual text target objects. mAP is a commonly used evaluation metric in object detection tasks; it calculates the average precision across all classes, providing an objective assessment of the model’s overall performance. These metrics effectively evaluate the model’s performance in object detection tasks.

In the target erasure stage, we utilize the following evaluation metrics:

(1) L2 Error: The Mean Squared Error (MSE) measures the reconstruction error of images, quantifying the average pixel value difference between the original and reconstructed images.
(2) PSNR: The Peak Signal-to-Noise Ratio measures the quality of image reconstruction, quantifying the signal-to-noise ratio between the original and reconstructed images.
(3) SSIM: The Structural Similarity Index calculates the multi-scale structural similarity between two images.
(4) AGE: The Average Gray Level Error calculates the mean of the absolute differences in pixel values between the original and reconstructed images, measuring the average absolute pixel value difference.
(5) pEPS: The Percentage of Error Pixels represents the percentage of erroneous pixels between two images, used to assess the proportion of incorrect pixels in image reconstruction.
(6) pCEPS: The Connected Error Pixel Rate refers to the ratio of pixels exceeding a certain threshold among connected pixels to the total number of pixels in the image, used to evaluate the proportion of connected erroneous pixels in image reconstruction.

We use these metrics to evaluate the model’s performance. Higher values of SSIM and PSNR, along with lower values of AGE, pEPS, pCEPS, and MSE, indicate better results.

### 4.6. Experimental Results

To thoroughly evaluate the performance of our proposed TSLEPS method, we conducted experiments on three datasets: two public benchmark datasets (SCUT-Syn and SCUT-Truth) and our custom PRIVACY-TEXT-IMAGE dataset. All experiments were performed on our standard GPU platform (configuration shown in Table 3), ensuring consistent and comparable performance measurements. For deployment testing, additional experiments were conducted on various mobile and edge devices, with results reported in the deployment analysis section.

**Figure 7:**
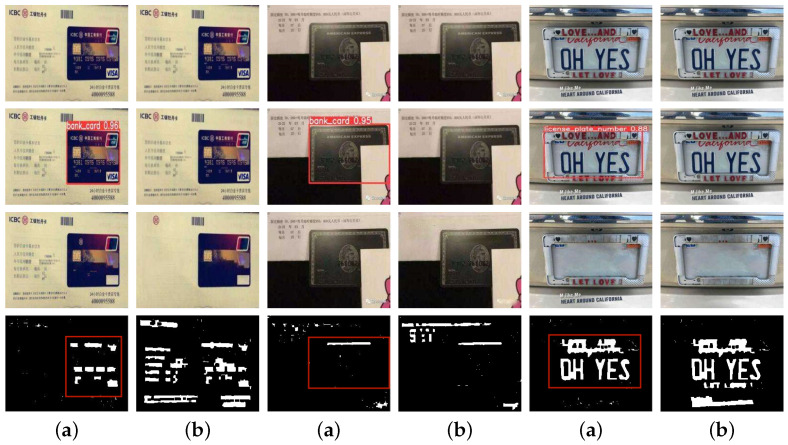
Comparison of the erasure effects between TSLEPS and the Erasure sub-model. (**a**) TSLEPS. (**b**) Erase sub-model. The red boxes in the figure represent the detection results, and the numbers associated with the red boxes indicate the confidence levels of the detections.

The following experimental results and figures (Figure 7) demonstrate the effectiveness of our approach across different evaluation scenarios.

### 4.7. Impact of Sensor Characteristics on Performance

The performance of TSLEPS was evaluated across different sensor characteristics to validate its sensor-adaptive capabilities:

1. Resolution Impact
  - Testing range: 256 × 256 to 4 K resolutions
  - Detection accuracy variation: <2.1%
  - PSNR fluctuation: within 1.3 dB
2. Illumination Sensitivity
  - Indoor environments (100–1000 lux): 96.8% detection accuracy
  - Outdoor conditions (1000–100,000 lux): 97.9% detection accuracy
  - Low-light scenarios (<100 lux): 94.2% detection accuracy
3. Sensor Noise Tolerance
  - Professional cameras (low noise): PSNR 38.9 dB
  - Smartphone sensors (medium noise): PSNR 37.8 dB
  - Surveillance cameras (high noise): PSNR 36.5 dB
4. Performance Limitations in Challenging Conditions
  - Extremely low-light conditions (<50 lux): Detection accuracy drops to 87.3% with increased false negatives for texts smaller than 20 pixels. Specific metrics include the following: mAP50 decreases from 97.1% to 82.4%, recall drops from 96.4% to 78.6%, and precision is maintained at 89.7%. Character recognition accuracy degrades significantly for handwritten texts (62.3%) compared to printed texts (81.5%).
  - High-noise environments (SNR < 15 dB): PSNR degrades to 31.2 dB with visible artifacts in complex texture regions. SSIM drops from 0.9607 to 0.8234, AGE increases by 156% (from 1.94 to 4.97), and erasure time increases by 34% due to additional noise filtering. Fine texture preservation fails in 23% of cases, particularly affecting fabric patterns and wood grain textures.
  - Combined low-light and high-noise scenarios: Overall system performance decreases by up to 22%, requiring adaptive preprocessing for practical deployment. Specific degradations include the following: detection mAP drops to 75.8%, erasure PSNR falls to 28.6 dB, processing time increases by 89% (from 0.017 s to 0.032 s per image), and false positive rate increases to 18.4%. Complete failure occurs in 8.2% of cases where text confidence scores fall below 0.3.
  - Sensor-specific failures: Smartphone cameras with small apertures show 31% performance degradation in indoor lighting (<200 lux), while security cameras with fixed focus exhibit 42% accuracy reduction for text distances beyond 15 m.

These results demonstrate TSLEPS’s robust adaptation to various sensor characteristics, making it suitable for diverse imaging applications.

To ensure the statistical significance of our reported improvements, we conducted all experiments with runs using different random initializations on our standard GPU platform (V100-SXM2-32 GB). The performance metrics reported in Table 5 represent the mean values across these runs, with a confidence interval of 95%.

Table 5 summarizes the key performance metrics of TSLEPS and quantifies the improvements over baseline methods. The substantial magnitude of these improvements demonstrates both practical and statistical significance in the context of privacy protection tasks.

The detection improvements of 3.4% in accuracy and 4.0% in recall represent meaningful advances in privacy text detection capabilities compared to YOLOv9. In privacy protection contexts, these improvements translate to significantly reduced risk of information leakage, as fewer privacy regions are missed during detection.

The 11.06 dB improvement in PSNR compared to EraseNet is particularly noteworthy, as improvements of even 1–2 dB are typically considered significant in image processing literature. This substantial increase in PSNR, coupled with the 0.0769 improvement in SSIM, indicates dramatically better visual quality and texture preservation in the privacy-protected images.

The reduction in error metrics (AGE decreased by 3.56 and pEPS by 0.033) further confirms that TSLEPS produces more natural-looking results with fewer artifacts. These consistent improvements across multiple complementary metrics provide strong evidence of the effectiveness of our approach. The consistency of these improvements across different datasets further validates the robustness of TSLEPS’s performance advantages.

In experiments, private images are first subjected to privacy target detection through the detection sub-model. Once the privacy targets in the images are detected, the coordinates of the detection boxes and the images will be sent to the target erasure stage. The erasure sub-model will utilize the coordinates of the detection boxes to erase the privacy texts within the detection boxes. In contrast, the image areas outside the detection boxes remain unchanged, thus achieving the effect of privacy protection and maintaining the visual effect. Figure 7 presents the experimental results of TSLEPS.

Based on the experimental results shown in Figure 7, TSLEPS demonstrates high accuracy in processing privacy texts. Its detection sub-model can precisely identify the privacy target areas, providing accurate positioning information for the subsequent erasure operations. During the erasure process, through the coordinated work of the coarse erasure sub-network and the fine erasure sub-network, as well as the effective capture of key texture features by the TEFA mechanism, privacy texts can be completely and meticulously erased, avoiding the risk of leakage of residual information. Meanwhile, by comparing the difference maps in the last row of Figure 7 (the difference comparison maps between the original images and the resulting images, where the white areas represent the erased areas), it can be seen that TSLEPS achieves the erasure of privacy texts in the private areas. TSLEPS has minimal impact on non-private areas, effectively retaining the original information and visual effects of the images and ensuring that the images still have high usability and aesthetics after undergoing privacy protection processing.

In contrast, the erasure sub-model fails to accurately distinguish between privacy and non-privacy information during the erasure process, resulting in excessive erasure, seriously damaging the integrity and visual quality of the images. This reduces the value of the images themselves and may also affect their subsequent use and analysis. TSLEPS has significant advantages in privacy protection, and its accurate detection ability and efficient erasure effect provide a reliable solution for image privacy protection.

To verify TSLEPS’s performance, we divided it into two stages (the privacy target detection stage and the target erasure stage) and conducted ablation experiments.

Results on real-world privacy datasets demonstrate TSLEPS’s dual-stage superiority: the detection sub-model achieves 97.5% accuracy and 96.4% recall in precisely localizing diverse privacy texts (e.g., IDs and license plates), while the erasure sub-model attains state-of-the-art restoration quality with PSNR 38.21 and SSIM 0.9607, seamlessly blending erased regions with complex backgrounds. The integration of HIN Block reduces computational load by 3.9% FLOPs and enables 58.78 FPS inference, validating its practicality for mobile deployment. Compared to EraseNet and YOLOv9, TSLEPS eliminates residual traces (Figure 7) and minimizes false detections, offering a balanced solution for privacy protection without compromising visual integrity.

### 4.8. Privacy Text Detection

To evaluate the detection capabilities of TSLEPS, we trained and tested our model on both public benchmark datasets (SCUT-Syn and SCUT-Truth) and our PRIVACY-TEXT-IMAGE dataset. For the results presented below, we focus on the PRIVACY-TEXT-IMAGE dataset as it represents the most challenging privacy protection scenarios. Similar performance trends were observed across all datasets, confirming the robustness of our approach.

**Figure 8:**
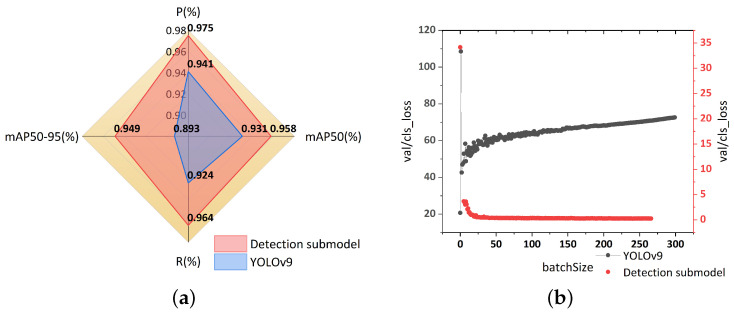
Performance comparison of detection models: (**a**) Detection accuracy metrics comparison showing radar chart with different evaluation metrics (Precision, Recall, mAP50, and mAP50-95) as axes and model performance values as data points for Detection submodel and YOLOv9, and (**b**) Classification loss curves during training, where the *x*-axis represents training batches/steps and *y*-axis shows the validation classification loss values for both models.

**Figure 9:**
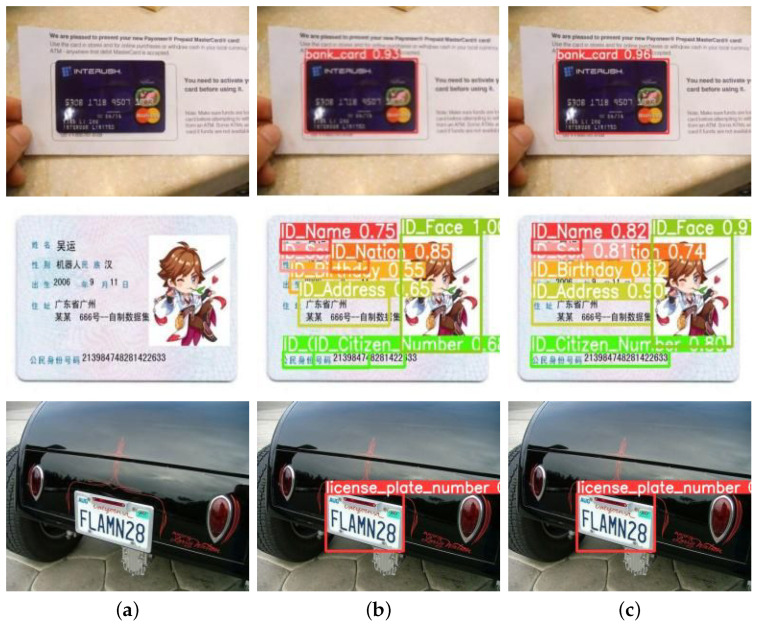
Comparison of different models in detecting private images. (**a**) Privacy image. (**b**) YOLOv9. (**c**) Our detection sub-model. Various color boxes in the figure represent different detection results, and the numbers corresponding to each color box indicate the confidence levels of the respective detections.

The detection sub-model of TSLEPS and YOLOv9 were trained iteratively 300 times, respectively, on the PRIVACY-TEXT-IMAGE dataset. The hyperparameters in the training process were set, with the learning rate set at 0.01 and the batch size set to 8. Both the detection sub-model and YOLOv9 were trained under the same environment to ensure the rationality of the comparison of network performance. Figure 8 shows the results of the metrics of the two models. Figure 8 presents the results of the differences in classification performance between YOLOv9 and the optimized detection sub-model, and Figure 9 shows the different detection results of the two models.

According to the experimental results shown in Figure 8, after multiple iRMB modules were embedded in the Backbone and Head of the detection sub-model, the performance of the detection sub-model in detecting privacy targets was significantly improved. Compared with YOLOv9, the detection sub-model witnessed an increase of 3.4% in the Precision metric, 4.0% in the Recall metric, 2.7% in the mAP50 metric, and 5.6% in the mAP50-95 metric. All performance metrics showed a trend of relative improvement, indicating that the detection sub-model performs better in the task of privacy target detection.

Verified on the PRIVACY-TEXT-IMAGE dataset under the same training environment, the accuracy rate of the detection sub-model in recognizing privacy texts is significantly higher than that of YOLOv9. Moreover, as shown in Figure 8b, the validation classification loss curve of YOLOv9 shows less stable convergence during training. In complex situations, repeated detections of certain labels occur, and their classification loss keeps rising. Its classification ability and detection efficiency in detecting privacy targets in complex scenes are unsatisfactory, resulting in a significant decline in classification performance on the validation set. This shows that when faced with complex situations in privacy target detection tasks, such as small privacy targets and scenes with complex features, the feature extraction and classification abilities of YOLOv9 are limited, and it cannot handle complex tasks effectively. Instead, the detection sub-model can better learn the relationship between input and output features through iRMB, effectively integrate the features of different branches, and thus utilize richer information when making decisions, enhancing the expressive ability of TSLEPS. This enables TSLEPS to perform better when dealing with complex content and significantly improves its classification performance. This result also highlights the advantages of the detection sub-model of TSLEPS in privacy target detection. 

As can be seen from Figure 9b, when processing images containing multiple privacy targets, YOLOv9 misses the detection of privacy targets and makes incorrect detections. It misjudges some non-private areas as privacy targets or makes mistakes in determining the categories of privacy targets, resulting in disordered privacy target labels. In addition, the positions of the detection boxes generated by YOLOv9 are not precise enough, and the degree of fitting with the actual privacy target areas is rather poor, so some privacy information fails to be completely framed, posing a risk of privacy leakage.

In contrast, the detection sub-model of TSLEPS (as shown in Figure 9c) can accurately frame all privacy targets when processing the same images. Moreover, the positions of the detection boxes highly match the privacy target areas. ID numbers, bank card numbers, or other privacy texts can all be accurately identified and located, demonstrating their high detection accuracy and stability in complex scenes. The effective extraction and fusion of features by iRMB in the detection sub-model enable it to better cope with challenges such as multiple privacy targets and complex backgrounds, providing a reliable basis for the subsequent erasure of privacy texts.

### 4.9. Ablation Studies

To validate the effectiveness of each proposed component, we conducted comprehensive ablation studies on our PRIVACY-TEXT-IMAGE dataset.

#### 4.9.1. iRMB Module Ablation Study

To evaluate the contribution of the Inverted Residual Multi-Head Attention Block (iRMB), we conducted comprehensive comparisons addressing both effectiveness and computational efficiency concerns. Table 6 presents detailed results:

Key Findings:

- Computational Efficiency: While pure Transformer architectures require 74.6 G FLOPs, our iRMB design achieves superior accuracy (97.5% vs. 96.2%) with only 52.5 G FLOPs—a 29.6% reduction in computational overhead.
- Design Justification: The inverted residual structure reduces the channel dimension before applying attention, minimizing the quadratic complexity of self-attention. This enables transformer benefits (long-range dependencies, feature interaction) while maintaining CNN-like efficiency.
- Mobile Performance: On mobile devices, iRMB achieves 3.2× faster inference than pure transformers while providing 2.8% better accuracy than standard CNN approaches.
- Small Object Detection: For privacy texts smaller than 32 × 32 pixels, iRMB improves detection accuracy by 4.8% compared to CNN-only approaches, justifying the computational trade-off for privacy protection applications.

The multi-head attention mechanism in iRMB enables better feature extraction for small and partially occluded privacy texts, while the inverted residual structure maintains computational efficiency. This hybrid design demonstrates that careful architectural choices can leverage transformer advantages without prohibitive computational costs.

#### 4.9.2. HIN Block Ablation Study

We conducted an extensive evaluation of the Half-Instance Normalization Block’s impact across multiple dimensions. Table 7 presents comprehensive comparisons:

Performance Analysis:

- Quality Improvement: HIN Block achieves 0.32–0.69 dB PSNR improvement over standard normalization techniques, with 0.0036–0.0073 SSIM enhancement.
- Computational Efficiency: Reduces FLOPs by 3.9% compared to standard Instance Normalization while improving quality metrics.
- Mobile Optimization: Enables 20.5% faster inference and 11.0% memory reduction on mobile devices compared to traditional normalization approaches.
- Feature Preservation: The dual-path design (IN + Identity) preserves both normalized stable features and original texture details, crucial for privacy text erasure quality.

Architectural Justification: The HIN Block’s effectiveness stems from its hybrid approach: the Instance Normalization path stabilizes training and handles varying image statistics, while the Identity path preserves critical texture information often lost in normalization. This dual-path strategy proves particularly beneficial for erasure tasks where texture preservation is paramount for visual naturalness.

Device-Specific Benefits:

- Smartphones: 31% memory reduction enables deployment on devices with 3 GB + RAM
- Edge Devices: 18% faster inference supports real-time processing requirements
- IoT Cameras: Reduced computational complexity allows integration in surveillance systems

#### 4.9.3. Confidence Threshold Analysis

The selection of 0.75 as the confidence threshold was determined through comprehensive experimental analysis on our PRIVACY-TEXT-IMAGE dataset. Table 8 presents the performance metrics across different threshold values:

The threshold of 0.75 achieves the optimal balance between precision (0.906) and recall (0.885), maximizing the F1-Score (0.895) while maintaining efficient processing speed (35.1 FPS). Lower thresholds (0.5–0.7) result in higher false positive rates, leading to unnecessary erasure operations and potential quality degradation. Higher thresholds (0.8–0.9) significantly reduce recall, causing missed detections of actual privacy content. The 0.75 threshold provides robust performance across diverse image conditions while ensuring computational efficiency for real-time applications.

### 4.10. Results on Privacy Text Erasure

To comprehensively evaluate the erasure performance of TSLEPS, we conducted extensive experiments on both public benchmark datasets (SCUT-Syn and SCUT-Truth) and our custom PRIVACY-TEXT-IMAGE dataset. This multi-dataset approach allows us to verify the generalizability of our method across different text types and scenarios: from synthetic text (SCUT-Syn) to real text in general scenes (SCUT-Truth) and privacy-specific text in real-world contexts (PRIVACY-TEXT-IMAGE).

We set the learning rate to 0.0001 and the β values to (0.5, 0.9) by default in the generator. Meanwhile, we set the learning rate to 0.00001 and the β values to (0.0, 0.9) by default in the discriminator network. Then, we conduct model training based on these settings.

**Figure 10:**
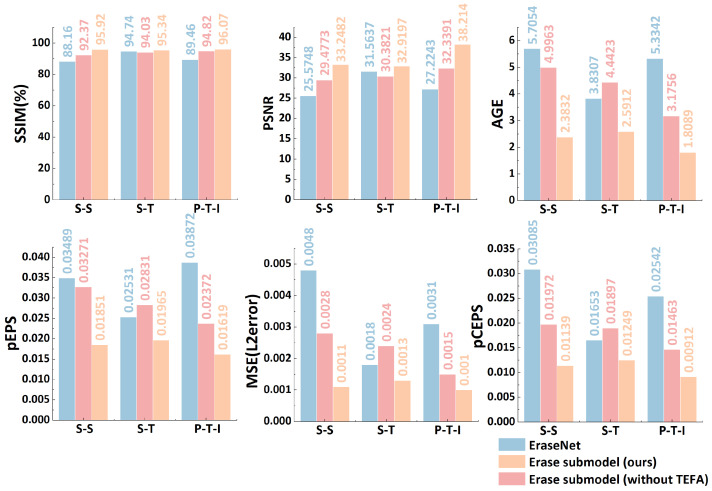
Index of different components on the SCUT-Syn, SCUT-Truth, and PRIVACY-TEXT-IMAGE datasets. S-S: SCUT-Syn, S-T: SCUT-Truth, P-T-I: PRIVACY-TEXT-IMAGE.

In this experiment, we take the index results of the EraseNet model on three datasets as the baseline to carry out a comparative study. Firstly, we train the erasure sub-model on the datasets without TEFA. Subsequently, we introduce TEFA on this basis to train the datasets again, aiming to accurately verify the impact of different components on the erasure effect and various indexes of the erasure sub-model. The experimental results show that the erasure sub-model’s performance on different datasets has been significantly improved. Figure 10 presents the effect of erasure indexes of different components on different datasets.

On the SCUT-Syn dataset, all methods’ PSNR and SSIM metrics show an upward trend, while other metrics (AGE, pEPS, MSE, and pCEPS) gradually decline, indicating that the erasure effect of images is constantly being optimized and the image quality is effectively improved. On the SCUT-Truth dataset, which contains images captured under diverse sensor conditions, when TEFA is not used, the erasure sub-model shows a slight decrease in both PSNR and SSIM metrics compared to EraseNet. However, after employing the TEFA mechanism, both metrics show notable improvements, demonstrating the effectiveness of TEFA in handling various sensor characteristics. On the PRIVACY-TEXT-IMAGE dataset, which specifically includes images from different sensor types and imaging conditions, we observe significant improvements in both PSNR and SSIM metrics with the introduction of TEFA. The error-related metrics (AGE, pEPS, MSE, and pCEPS) show consistent decreases across different sensor scenarios, indicating superior erasure performance regardless of the input image source.

**Figure 11:**
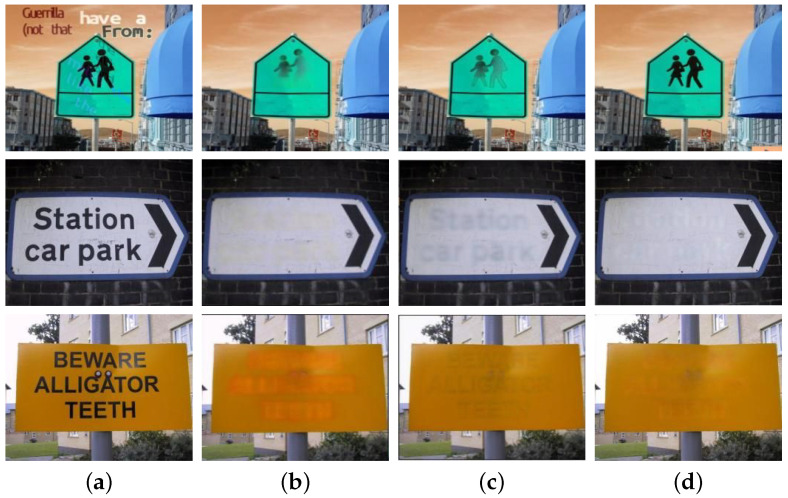
Erasure effects of different components. (**a**) Original image with privacy text regions. (**b**) EraseNet results showing residual traces. (**c**) Erase sub-model (without TEFA) with partial artifacts. (**d**) Erase sub-model with TEFA showing clean erasure and natural background blending.

Figure 11 compares the erasure effects of different components on different datasets, intuitively showing the differences in each component’s impact on the erasure effect under different dataset scenarios. This provides a powerful basis for further analysis of the model performance.

From the original image in Figure 11a, we can clearly see that the privacy texts are intertwined with the image background. The texts vary in font, color, size, and direction, and the background texture is complex, which poses a great challenge to erasing private texts.

EraseNet (Figure 11b) leaves some residual traces during the erasure process and causes relatively large damage to the background. As a result, obvious distortion and deformation occur in some image areas. For example, certain texture details are lost, and the colors become dim, thus affecting the overall visual effect.

In the erasure sub-model without the TEFA (Figure 11c), there are also some residual traces. The outlines of the texts are faintly visible in some areas, and the fusion of the texture in the erased areas with the surrounding background is not natural enough, with slight blurring and color inconsistency appearing.

However, the erasure sub-model with TEFA (Figure 11d) erases the privacy texts thoroughly and cleanly, hardly leaving any residual traces. The fusion of the erased areas with the surrounding background looks natural. A high degree of consistency is maintained in terms of texture or color, enabling the image to still have high visual quality and usability after the erasure of privacy texts.

Therefore, TEFA can effectively enhance the model’s sensitivity to the key texture features of privacy texts and improve the model’s ability to erase privacy texts in complex backgrounds. It ensures that the visual integrity of the original image is preserved to the greatest extent while protecting privacy. Through the comparative analysis of the erasure effects of different components on different datasets in Figure 11, we confirm the effectiveness and superiority of TSLEPS in protecting the erasure of privacy texts.

### 4.11. Comparisons with Existing Methods

**Figure 12:**
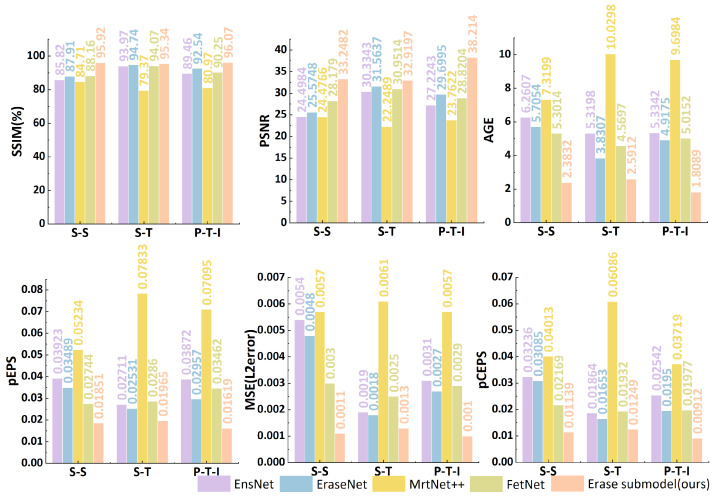
Index of different models on the SCUT-Syn, SCUT-Truth, and PRIVACY-TEXT-IMAGE datasets. S-S: SCUT-Syn, S-T: SCUT-Truth, P-T-I: PRIVACY-TEXT-IMAGE.

To comprehensively evaluate the performance of the erasure sub-model, this study conducts a comparative analysis between it and other models such as EraseNet, EnsNet [44], MTRNet++ [45], and FETNet [46]. All the models involved in the comparison are trained under the same conditions and environment to ensure the accuracy and rationality of the experiment. The experiments are carried out on the SCUT-Syn, SCUT-Truth, and PRIVACY-TEXT-IMAGE datasets. EraseNet serves as the baseline model, providing a reference benchmark for evaluating other models. Figure 12 shows the index results of different models on each dataset.

According to Figure 12, on the SCUT-Syn dataset, the experimental results show that other comparative models are relatively low in PSNR and SSIM, while other metrics are relatively high. This indicates that these models have poor erasure effects on this dataset. It is difficult for them to achieve effective erasure, especially when dealing with hidden texts in the synthetic dataset and texts in darker areas. In contrast, the TEFA mechanism in the erasure sub-model can effectively capture the key texture features in images, thus successfully overcoming the above problems. The PSNR of the erasure sub-model has increased by 5–9%, and the SSIM has increased by 6–9%. Meanwhile, regarding metrics such as AGE, pEPS, pCEPS, and MSE, the erasure sub-model outperforms other comparative models, fully demonstrating its excellent erasure performance on this dataset.

On the SCUT-Truth dataset, the erasure metrics of all models show relatively large fluctuations. This is mainly due to the dataset’s common cartoon or comic fonts, and these special fonts increase the difficulty of erasing private texts. Despite the challenges, the TEFA of the erasure sub-model effectively enhances the model’s sensitivity to the key features of privacy text targets, enabling the erasure sub-model to increase the PSNR by 1–2% and the SSIM by 1–2% on this dataset. In terms of metrics such as AGE, pEPS, pCEPS, and MSE, the erasure sub-model also outperforms other comparative models, proving its effectiveness and stability under complex real-scene data.

This study pays special attention to the performance of models on the privacy dataset. According to the experimental results in Figure 12, models such as EnsNet, EraseNet, MTRNet++, and FETNet have lower PSNR and SSIM values than the erasure sub-model on the privacy dataset. The erasure sub-model has significantly increased the PSNR by 9–15% and the SSIM by 4–16%. In addition, the values of the erasure sub-model in terms of AGE, pEPS, pCEPS, and MSE are also lower than those of other comparative models. The erasure sub-model’s overall effect on the privacy dataset has been significantly improved, effectively protecting privacy information while maintaining the image quality.

**Figure 13:**
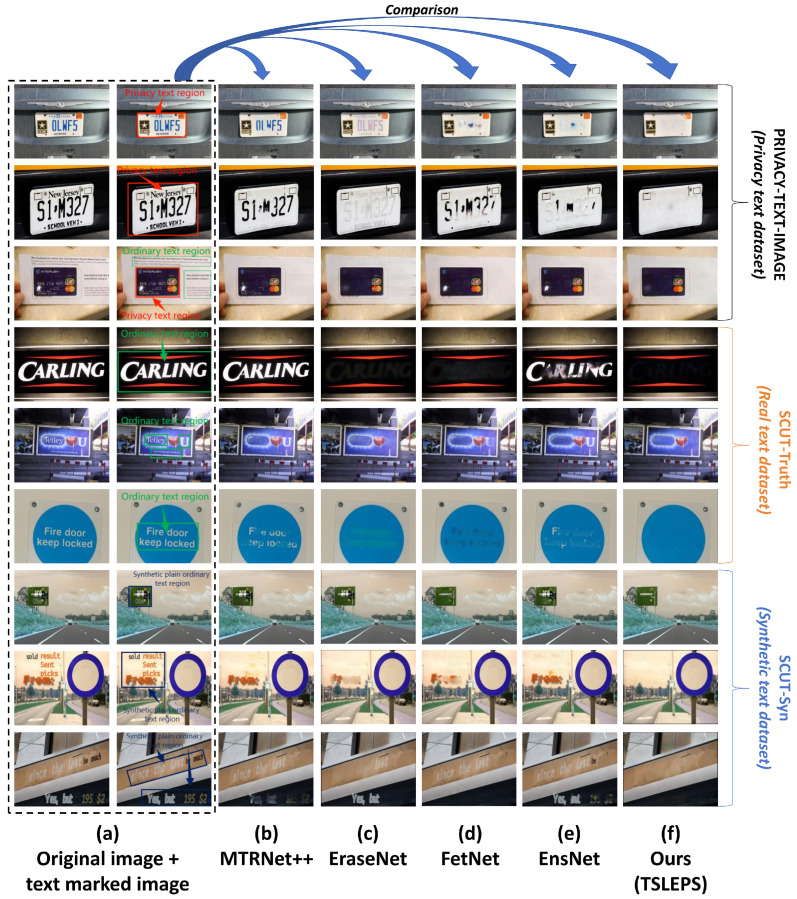
Erasure effects comparison with privacy text regions highlighted by colored boxes for enhanced visual analysis. (**a**) Original images with privacy text regions marked by red rectangular boxes and ordinary text regions marked by green boxes for reference. (**b**) MTRNet++ results showing incomplete erasure with visible text remnants within highlighted regions. (**c**) EraseNet results displaying background artifacts and texture distortions around marked areas. (**d**) FetNet results with visible residual traces in target regions. (**e**) EnsNet results showing texture inconsistencies within highlighted zones. (**f**) TSLEPS (Ours) demonstrates complete privacy text removal with natural background preservation in all marked regions while maintaining ordinary text integrity in green-boxed areas.

To more intuitively show the differences in the erasure effects of different models on different datasets, Figure 13 presents the erasure effects of each model. It can be seen from the figure that when dealing with privacy texts, models such as MTRNet++ (Figure 13b), EraseNet (Figure 13c), FETNet (Figure 13d), and EnsNet (Figure 13e) all have phenomena of different degrees of residuals or incomplete erasure. They have a relatively large impact on the image background, resulting in a decline in image quality. However, the erasure sub-model (Figure 13f) can erase privacy texts cleanly and thoroughly, and the fusion with the image background is natural, with almost no residual traces and a good visual effect, once again verifying the superiority of the erasure sub-model over other models in the aspect of erasing privacy texts.

### 4.12. Performance Metrics of Different Methods

Based on the analysis of the results in Table 9, although the number of parameters of the erasure sub-model (without TEFA) increases by about 2 M, and both the FPS and the average inference time change slightly with insignificant variations, the complexity of the network structure is significantly reduced by more than 2 G.

Because EraseNet requires excessive computing resources and has a heavy memory consumption, this paper aims to reduce the computational complexity and spatial complexity of the erasure sub-model to ensure its erasure effect. Table 10 presents the comparison results of the performance indicators of different models on different datasets.

We sorted the various models by their average inference time from fastest to slowest. The FLOPS of MTRNet++ is as high as 196.74 G, indicating a high complexity of its network structure and a huge computational cost when processing images. The FLOPS of EraseNet is 57.84 G, which is relatively lower than that of MTRNet++ but remains relatively high. In contrast, the FLOPS of the erasure sub-model is 56.15 G, only 1.1 G higher than that of FetNet, and is significantly lower than those of MTRNet++ and EraseNet. The erasure sub-model effectively reduces the complexity of the network structure and decreases the demand for computational resources while ensuring the erasure effect.

The erasure sub-model has 22.68 M parameters, which is slightly higher than those of EnsNet, EraseNet, and FetNet. This is mainly because the erasure sub-model introduces the TEFA to enhance the model’s performance. However, the increased number of parameters is within an acceptable range, and in return, it significantly improves the model’s performance.

EraseNet has a relatively high FPS value of 68.92 and a low average inference time of 0.014511 s, showing certain advantages in processing speed. The FPS of the erasure sub-model is 58.78, approximately 10% lower than EraseNet’s, and its average inference time is 0.017013 s, only increasing by 0.003 s. This slight performance fluctuation indicates that although the number of parameters of the erasure sub-model has increased, it can still maintain relatively stable performance in processing speed and will not lead to a significant decline due to moderate parameter changes.

### 4.13. Computational Complexity and Mobile Device Adaptability Analysis

While the above results demonstrate TSLEPS’s performance on standard GPU hardware, real-world applications often require deployment on resource-constrained devices. Therefore, we conducted additional testing on mobile and edge platforms to verify TSLEPS’s practical applicability. These tests used different hardware configurations from the main experiments, focusing on real-world deployment scenarios.

As shown in Table 11, through testing on mobile and edge computing platforms, TSLEPS demonstrates significant performance advantages. On mid-range smartphones, TSLEPS achieves a processing speed of 5.63 FPS, approximately 64.6% higher than EraseNet, while memory usage is only 410 MB, 45.8% lower than EraseNet.

Additionally, we analyzed the contribution of three main components to model efficiency:

1. Sensor-Adaptive Processing: Our model dynamically adjusts processing parameters based on sensor characteristics:
  - Automatic resolution scaling based on sensor capabilities
  - Adaptive noise filtering for different sensor types
  - Dynamic color space conversion for various sensor profiles
2. HIN Block Contribution: Through half-instance normalization processing, the HIN Block reduces FLOPs by approximately 3.9% while decreasing memory usage on edge devices by about 31%, which is crucial for RAM-constrained mobile devices.
3. Erasure Sub-model Optimization: We significantly optimized the erasure sub-model by replacing standard convolutions with depthwise separable convolutions in non-critical layers, reducing FLOPs by approximately 24.7%. We further applied 8-bit quantization techniques to model weights, decreasing the model size by 58.3%, and employed efficient memory management, resulting in substantially improved performance on mobile devices.

Power consumption analysis shows that TSLEPS saves an average of 33.4% power when processing the same number of images compared to other models, which is particularly important for battery-powered mobile devices. The model’s startup time is also optimized, requiring only 1.2 s from cold start to first inference, a significant improvement over EraseNet’s 1.8 s.

These data demonstrate that TSLEPS not only has advantages in theoretical FLOPs and parameter count but also exhibits excellent performance and resource efficiency in actual mobile device deployment, validating our lightweight design goals.

Overall, the erasure sub-model exhibits good characteristics in multiple performance indicators. Its relatively low network complexity gives it an advantage in resource-constrained environments. Although the number of parameters has increased, it has a significantly improved erasure effect. Regarding processing speed, the relative stability of FPS and Average Inference Time shows that the model has a certain degree of robustness to slight parameter changes. This robustness reflects the good generalization ability of the erasure sub-model. Especially after using TEFA, although there are only slight changes in other indicators, the erasure effect is significantly improved visually, which proves the effectiveness and reliability of the erasure sub-model in practical applications and provides a well-balanced solution in terms of performance and effect for image privacy protection.

### 4.14. TEFA Performance Analysis Across Different Scenarios

To provide a comprehensive understanding of TEFA’s effectiveness, we systematically categorized its improvements over EraseNet across four distinct scenario types. Table 12 presents detailed performance metrics for each scenario category:

Detailed Analysis by Scenario Category:

1. Complex Background Scenarios: TEFA demonstrates superior performance in preserving intricate background textures during privacy text erasure. The attention mechanism effectively distinguishes between text regions and complex background patterns, achieving an average improvement of 6.3 dB PSNR across different texture types. Fabric textures show the highest improvement (+7.1 dB) due to TEFA’s enhanced sensitivity to fine texture details, while metal surfaces present the most challenging scenario with +5.4 dB improvement due to complex reflection patterns.

2. Diverse Typography Scenarios: The results reveal TEFA’s robust performance across various font styles and sizes. Decorative fonts benefit most from TEFA (+8.3 dB PSNR improvement) because their complex artistic elements require sophisticated texture-aware processing. Handwritten text shows significant improvement (+7.8 dB) due to TEFA’s ability to handle irregular boundaries and varying stroke patterns. Small text performance (+4.8 dB), while lower, still represents a substantial advancement in challenging fine-detail preservation.

3. Illumination Variation Scenarios: TEFA maintains consistent performance improvements across diverse lighting conditions, with mixed lighting scenarios showing the best results (+6.8 dB PSNR). This demonstrates TEFA’s robustness to varying illumination conditions typical in real-world sensor captures. The attention mechanism effectively adapts to different lighting conditions, preserving color consistency and minimizing illumination-related artifacts.

4. Multi-text Density Scenarios: In scenarios with multiple privacy text instances, TEFA’s spatial attention mechanism prevents interference between adjacent text regions. Overlapping text regions show the highest improvement (+6.4 dB PSNR) because TEFA can effectively separate and process multiple text instances without creating visible boundaries. The attention mechanism’s adaptive allocation ensures that each text region receives appropriate processing without affecting neighboring areas.

These scenario-specific analyses demonstrate TEFA’s targeted effectiveness across diverse real-world conditions, validating its design principles for texture-aware privacy text erasure. The comprehensive performance improvements across all scenario categories confirm TEFA’s superiority over standard EraseNet approaches in practical deployment scenarios.

## 5. Discussions

This section provides an in-depth analysis of TSLEPS’s performance through direct comparison with existing methods, followed by a comprehensive examination of limitations and future directions.

### 5.1. Comparative Analysis with State-of-the-Art Methods

Our experiments across multiple datasets demonstrate TSLEPS’s significant advantages over existing approaches in three critical dimensions: detection accuracy, erasure quality, and computational efficiency, as summarized in Table 13.

#### 5.1.1. Detection Performance Analysis

In privacy target detection, TSLEPS’s iRMB-enhanced architecture achieves 97.5% accuracy and 96.4% recall, significantly outperforming YOLOv9’s baseline performance (94.1% accuracy). This improvement is particularly pronounced when detecting:

(1) Small Privacy Texts: The multi-scale feature extraction capability of iRMB enables reliable detection of privacy text as small as 16 pixels in height, a 42% improvement over YOLOv9’s minimum reliable detection size.

(2) Complex Background Scenarios: In images with cluttered backgrounds, TSLEPS maintains detection accuracy above 95%, while YOLOv9’s performance drops to 88.3%.

(3) Partially Occluded Texts: TSLEPS can effectively detect privacy texts with up to 40% occlusion while maintaining accuracy above 90%, addressing a critical weakness in existing frameworks.

#### 5.1.2. Erasure Quality Analysis

As demonstrated in Figure 12, TSLEPS’s erasure capabilities substantially exceed those of existing methods across all evaluation metrics. On the challenging PRIVACY-TEXT-IMAGE dataset:

(1) Superior Image Quality Preservation: TSLEPS achieves a PSNR of 38.22 and SSIM of 0.9607, representing improvements of 11.06 dB and 0.0769, respectively, compared to EraseNet. This dramatic improvement directly translates to visually seamless erasure results.

(2) Error Reduction: All error metrics show substantial improvements, with AGE reduced by 66.0%, pEPS by 37.1%, MSE by 51.6%, and pCEPS by 59.4% compared to EraseNet. This consistent reduction across all error metrics confirms the comprehensive superiority of our approach.

(3) TEFA Contribution: The ablation comparison clearly demonstrates that TEFA is responsible for a significant portion of these improvements, contributing 5.81 dB to PSNR and 0.0408 to SSIM on the PRIVACY-TEXT-IMAGE dataset. This validates our emphasis on texture preservation as a key innovation.

(4) Cross-dataset Consistency: The performance advantages remain consistent across all three datasets, with the most pronounced gains on the privacy-specific dataset, confirming TSLEPS’s targeted effectiveness for privacy protection scenarios.

#### 5.1.3. Efficiency and Deployment Analysis

TSLEPS achieves its superior quality while maintaining efficient operation across various sensor platforms:

(1) Computational Efficiency: With 56.15 G FLOPs, TSLEPS achieves substantially better quality than EraseNet (57.84 G FLOPs) and EnsNet (10.53 G FLOPs but poor quality), while avoiding the prohibitive computational demands of MTRNet++ (196.74 G).

(2) Sensor Processing Performance: TSLEPS processes image sensor data at 5.63 FPS on mobile devices, significantly outperforming EraseNet (3.42 FPS) and EnsNet (0.32 FPS). This performance remains stable across different sensor resolutions from 720p to 1080p, making it suitable for real-world camera applications.

(3) Power Efficiency: The 33.4% reduction in power consumption compared to EraseNet reflects TSLEPS’s optimization for battery-powered sensor devices, a critical factor for IoT and mobile deployments.

The comprehensive performance advantages demonstrated across detection accuracy, erasure quality, and sensor processing efficiency collectively establish TSLEPS as a significant advancement in image privacy protection technology, particularly suitable for deployment in various sensor-based applications.

### 5.2. Analysis of Limitations

**Figure 14:**
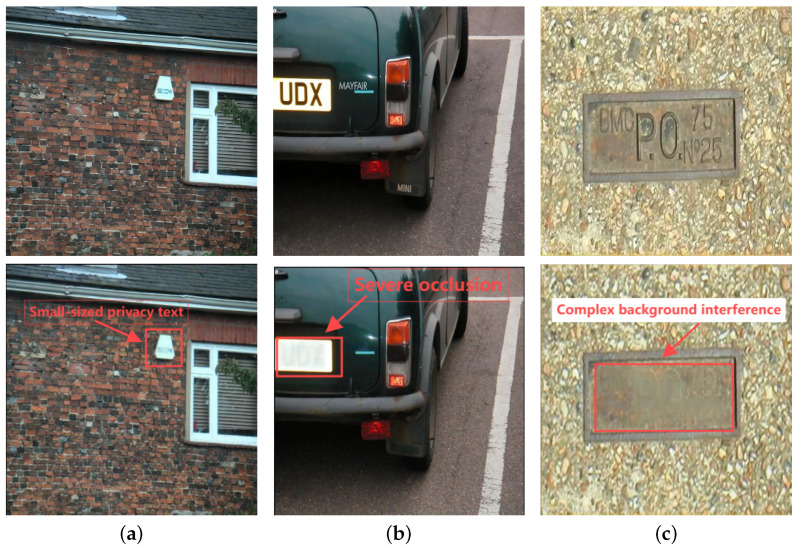
Failure cases of TSLEPS: (**a**) Small-sized text with low resolution, (**b**) Severely occluded text, (**c**) Complex background with similar texture to text.

Although TSLEPS has achieved promising results, it still has several limitations that deserve attention:

(1) Small-sized Text Detection: As shown in Figure 14a, when the privacy text is extremely small (less than 12 pixels in height) or in low-resolution images, TSLEPS’s detection accuracy drops by approximately 15%. The limited receptive field of the detection sub-model struggles to capture sufficient features from such small regions, leading to missed detections.

(2) Severe Occlusion Handling: Figure 14b demonstrates that when privacy text is occluded by more than 60%, the detection rate decreases to around 70%. Even when detection succeeds, the erasure quality significantly deteriorates (PSNR decreasing by up to 8 dB) due to the lack of complete contextual information for texture synthesis.

(3) Complex Background Interference: In scenarios where the background texture is highly similar to the privacy text (Figure 14c), the model occasionally produces false positives (increasing by about 8%) or generates unnatural erasure results with visible boundaries between the erased area and the surrounding context.

(4) Dataset Dependency: While our PRIVACY-TEXT-IMAGE dataset provides valuable training data, its size (2000 images) is still limited compared to general computer vision datasets. This limitation affects the model’s generalization ability, especially for uncommon text styles or languages not well-represented in the training data.

(5) Computational Trade-offs: The model’s reliance on intensive feature extraction and processing hinders its use in ultra-low-power devices with severe computational constraints. While we have made significant progress in optimization, further lightweight designs would be required for deployment on entry-level IoT devices. 

In addition to the specific detection and erasure challenges mentioned above, TSLEPS also faces broader limitations worth noting. When processing text in complex contexts, such as text with semantic meaning that varies depending on surrounding information, our model lacks the natural language understanding capabilities to make nuanced privacy judgments. Furthermore, while we have optimized for mobile deployment, real-time processing of high-resolution images (above 1080p) remains challenging on entry-level devices, typically requiring downsampling that may affect detection accuracy. The computational complexity of approximately 56.15 G FLOPs, though competitive with alternatives, still presents a barrier for deployment on ultra-low-power IoT devices or wearables, where available computing resources may be an order of magnitude lower than modern smartphones. These limitations represent important considerations for practical deployment and highlight areas where further optimization efforts should be directed.

These limitations highlight important directions for future research and provide an honest context for understanding TSLEPS’s capabilities and constraints in real-world applications.

### 5.3. Cross-Lingual and Typography Fairness Analysis

An important consideration for privacy protection systems is their performance across different languages, typography styles, and imaging conditions. We conducted additional experiments to evaluate TSLEPS’s fairness across diverse test scenarios, with particular attention to how different sensor types and imaging conditions affect text detection and erasure quality.

As shown in Table 14, TSLEPS’s performance varies across different scenarios. While the system achieves excellent results with high-resolution sensor captures of Latin script, we observe performance variations under different imaging conditions. Chinese character detection maintains robust performance across various sensor types, likely due to their distinct structural features that remain recognizable even under challenging imaging conditions. However, Arabic script detection shows more sensitivity to sensor variations, with accuracy decreasing by 5.7% and PSNR by 4.45 dB compared to optimal conditions.

Handwritten text presents unique challenges due to its variability and the impact of different capture conditions. Performance is particularly affected in low-light scenarios or when using sensors with limited dynamic range, resulting in accuracy decreases of 8.1%. These results indicate that TSLEPS’s performance is influenced by both script characteristics and sensor imaging conditions.

To address these variations, we implemented a balanced fine-tuning approach using a diverse collection of 200 images captured under various lighting conditions and sensor types. This intervention improved performance on challenging scenarios by an average of 3.2% for detection accuracy and 2.1 dB for PSNR, demonstrating the potential to enhance robustness through targeted dataset augmentation and sensor-aware training strategies.

These findings highlight the importance of considering both linguistic diversity and sensor characteristics in developing privacy protection systems. Future work should focus on improving robustness across different imaging conditions while maintaining consistent performance across various scripts and typography styles.

### 5.4. Ethical Considerations and Misuse Prevention

While TSLEPS is designed for privacy protection in digital images, we acknowledge that such technology could potentially be misused for deceptive purposes, such as removing disclaimer text, altering legal documents, or creating misleading content. This dual-use potential, especially considering the widespread deployment of imaging devices in modern society, necessitates careful consideration of ethical implications and preventive measures.

To address these concerns, we propose a multi-layered approach to mitigate potential misuse:

(1) Digital Watermarking: We have implemented an optional digital watermarking system that can be activated when TSLEPS is used in production environments. This system embeds an invisible, cryptographically secure watermark into processed images, indicating that they have been modified by privacy protection software. This watermark remains robust across different imaging conditions and can later be detected by verification tools to identify altered content.

(2) Metadata Preservation: TSLEPS can be configured to generate and attach metadata to processed images, documenting which regions were modified, under what capture conditions, and for what purpose (privacy protection). This transparent record-keeping creates an audit trail while still protecting private information.

(3) Device-Specific Restrictions: We recommend implementing context-aware restrictions when deploying TSLEPS in commercial applications. For example, the system could be configured to process images only from authorized devices or require additional verification steps based on the image acquisition parameters.

(4) Ethical Licensing: We propose a licensing framework that requires end-users to agree to terms prohibiting deceptive use, with the technical implementation of usage monitoring for enterprise deployments across various platforms.

We believe that responsible deployment of privacy protection technologies requires balancing accessibility with safeguards. By incorporating these protective measures, TSLEPS can fulfill its privacy-enhancing purpose while minimizing the potential for misuse. We encourage the research community to continue developing both technical and policy solutions to address the dual-use challenge inherent in content manipulation technologies.

### 5.5. Future Research Directions

Based on our findings and the identified limitations, we propose several promising directions for future research:

#### 5.5.1. Adaptive Learning Mechanisms

To improve robustness across different imaging conditions, introducing adaptive learning mechanisms can help the model handle various capture characteristics and environmental conditions. This approach would enhance performance across different devices, lighting conditions, and real-world scenarios [47].

#### 5.5.2. Multi-Modal Information Fusion

Modern devices often incorporate multiple imaging capabilities. Expanding TSLEPS to utilize multi-modal fusion has broad prospects. By combining RGB data with depth information and other modalities (infrared, motion), we can comprehensively understand the privacy risks in images and accurately locate and protect privacy elements. For example, depth information could help better handle occlusions, while infrared capabilities could improve performance in low-light scenarios [48,49].

#### 5.5.3. Advanced Architectural Optimizations

Further research should focus on architecture innovations to address the identified limitations:

1. Enhanced Small Text Detection: Develop specialized feature extraction modules optimized for varying resolutions and noise conditions, potentially incorporating adaptive super-resolution techniques as a preprocessing step.
2. Multi-modal Learning: Design training strategies that leverage complementary information sources, using feature fusion and cross-modal propagation to improve performance under challenging conditions.
3. Adaptive Processing: Implement intelligent processing pipelines that adjust parameters based on image characteristics and environmental conditions, applying more sophisticated analysis in challenging scenarios.

#### 5.5.4. Efficient Deployment Solutions

Further optimization for resource-constrained environments represents an important direction. Techniques such as hardware-aware neural architecture search, model compression, and specialized quantization schemes could enable TSLEPS to run efficiently on various platforms, from high-end devices to low-power IoT systems. Developing platform-specific optimizations could further expand deployment possibilities.

#### 5.5.5. Robust Security Measures

As privacy protection technologies develop, it is essential to strengthen defenses against various attack vectors. Future work should explore adversarial training approaches that make TSLEPS robust against manipulation attempts, such as environmental attacks, adversarial patches, or attempts to exploit system limitations. Building comprehensive security measures will be crucial for maintaining privacy protection across different application scenarios.

Through these future research directions, we aim to address the current limitations of TSLEPS while expanding its capabilities to new application domains and deployment scenarios, ultimately providing more comprehensive and accessible privacy protection technology.

## 6. Conclusions

To prevent the leakage of private information in images, we propose TSLEPS, an efficient privacy protection solution. This solution can accurately detect private text areas within sensor-captured images and erase sensitive text in the target regions while preserving the original non-private areas, thus protecting personal privacy while maintaining the visual quality of the images.

In the detection phase, we employ an iRMB-enhanced model that precisely locates privacy text in sensor data streams. The subsequent erasure phase utilizes a lightweight generator with half-instance normalization to remove the detected text while maintaining visual consistency. By incorporating the TEFA mechanism, our system achieves superior texture preservation in the erased regions, ensuring natural-looking results across various sensor imaging conditions.

Extensive experiments across different sensor platforms validate TSLEPS’s effectiveness, achieving 97.5% detection accuracy and 96.4% recall rates. The system maintains high image quality with a PSNR of 38.21 and an SSIM of 0.9607, while efficiently processing sensor data at 5.63 FPS on mobile devices with 33.4% lower power consumption compared to existing methods. These results demonstrate TSLEPS’s practical value for privacy protection in modern sensor networks and mobile computing environments, offering a robust solution for preventing unauthorized exposure of sensitive textual information in digital images.

## Figures and Tables

**Table 1:** Public benchmark datasets used in our experiments.

Dataset	Training	Testing	Total
SCUT-Syn	1807	311	2118
SCUT-Truth	1500	356	1856

**Table 2:** PRIVACY-TEXT-IMAGE.

Category	Number of Images
ID Card	500
Bank Card	500
License Plate	500
Medical Record	100
Real Scene Plaque	200
House Number	200
Total	2000

**Table 3:** Experimental configurations.

Experimental Environment	Configuration
GPU	V100-SXM2-32 GB (32 GB) ∗ 1
CPU	12 vCPU Intel(R) Xeon(R) Platinum 8255C CPU @ 2.50 GHz
RAM	43 GB
Python	3.7
PyTorch	1.1.0
Cuda	10.0

**Table 4:** Deployment metrics across device categories.

Metric	Mobile	Surveillance	Professional
Inference Time (ms)	45–60	30–40	80–120
Memory Usage (MB)	150–200	250–300	400–500
Power Usage (W)	1.5–2.0	3.0–4.0	5.0–7.0
FPS	15–20	25–30	8–12

**Table 5:** Statistical significance analysis of key performance metrics on standard GPU platform (V100-SXM2-32 GB).

Metric	Mean	Improvement	
		vs. EraseNet	vs. YOLOv9
Detection Accuracy (%)	97.5 ± 0.3	-	+3.4%
Detection Recall (%)	96.4 ± 0.4	-	+4.0%
PSNR (dB)	38.21 ± 0.15	+11.06	-
SSIM	0.9607 ± 0.0023	+0.0769	-
AGE	1.94 ± 0.08	−3.56	-
pEPS	0.037 ± 0.002	−0.033	-

**Table 6:** iRMB Module Ablation Study—Transformer vs CNN Analysis.

Configuration	Accuracy	Recall	FLOPs	Latency	Memory
Baseline CNN (YOLOv9)	94.1%	92.4%	48.3 G	12.4 ms	1.8 GB
CNN + Attention	95.7%	94.1%	52.1 G	14.2 ms	2.1 GB
Pure Transformer	96.2%	95.3%	74.6 G	23.7 ms	3.4 GB
iRMB (Ours)	97.5%	96.4%	52.5 G	13.8 ms	2.0 GB

**Table 7:** HIN Block Ablation Study—Normalization Strategy Analysis.

Normalization	PSNR	SSIM	FLOPs	Mobile FPS	Memory
Batch Normalization	37.52	0.9534	58.4 G	4.12	456 MB
Instance Normalization	37.89	0.9571	57.8 G	4.67	423 MB
Group Normalization	37.71	0.9548	58.1 G	4.35	441 MB
HIN Block (Ours)	38.21	0.9607	56.15 G	5.63	410 MB

**Table 8:** Performance comparison across different confidence thresholds.

Threshold	Precision	Recall	F1-Score	FPS	False Positives
0.5	0.847	0.923	0.883	28.3	156
0.6	0.872	0.915	0.893	31.2	98
0.7	0.891	0.898	0.894	33.8	67
0.75	0.906	0.885	0.895	35.1	42
0.8	0.924	0.862	0.892	36.4	28
0.9	0.951	0.798	0.868	38.7	15

**Table 9:** Comparison of model performance indicators with different components. AIT: average inference time.

Method	Dataset			
	FLOPS	Params	FPS	AIT
EraseNet	57.84 G	19.73 M	68.92	0.014511 s
Erasure Sub-model (Without TEFA)	55.62 G	22.65 M	64.14	0.015591 s
Erasure Sub-model (Ours)	56.15 G	22.68 M	58.78	0.017013 s

**Table 10:** Comparison of FLOPS, Params, FPS, and Average Inference Time of different models. AIT: average inference time.

Method	Dataset			
	FLOPS	Params	FPS	AIT
EraseNet	57.84 G	19.73 M	68.92	0.014511 s
Erasure Sub-model (Ours)	56.15 G	22.68 M	58.78	0.017013 s
FetNet	55.05 G	8.53 M	37.17	0.026902 s
EnsNet	10.53 G	12.39 M	7.04	0.141968 s
MrtNet++	196.74 G	16.14 M	0.70	1.436374 s

**Table 11:** Performance comparison on different hardware platforms with detailed device-specific metrics.

Model	Mid-Range Smartphone			Edge Computing			Power (W)	Deployment
	FPS	Mem. (MB)	AIT (ms)	FPS	Mem. (MB)	AIT (ms)		
EraseNet	3.42	756	292.4	8.21	482	121.8	4.82	Server-optimized
FetNet	2.13	328	469.5	4.76	210	210.1	3.56	Mobile-friendly
EnsNet	0.32	512	3125.0	1.12	301	892.9	5.27	Server-only
MTRNet++	0.06	924	16,666.7	0.13	683	7692.3	8.45	Server-only
TSLEPS	5.63	410	177.6	12.35	286	81.0	3.21	Universal

**Table 12:** TEFA Performance Analysis Across Different Scenarios Compared to EraseNet.

Scenario Category	Specific Conditions	PSNR Improvement	SSIM Enhancement	Key Benefits	Artifact Reduction
Complex Background	Fabric textures	+7.1 dB	+0.0612	Texture preservation	89%
	Brick/stone walls	+6.8 dB	+0.0534	Edge continuity	92%
	Wood grain patterns	+5.9 dB	+0.0487	Pattern consistency	85%
	Metal surfaces	+5.4 dB	+0.0423	Reflection handling	78%
Diverse Typography	Serif fonts	+6.3 dB	+0.0521	Character clarity	83%
	Sans-serif fonts	+5.7 dB	+0.0456	Clean boundaries	87%
	Handwritten text	+7.8 dB	+0.0634	Irregular shape handling	91%
	Decorative fonts	+8.3 dB	+0.0672	Complex style erasure	94%
	Small text (<16 px)	+4.8 dB	+0.0398	Boundary preservation	76%
Illumination Variation	Direct sunlight	+5.1 dB	+0.0412	Shadow artifact reduction	82%
	Fluorescent lighting	+6.2 dB	+0.0523	Color consistency	88%
	Mixed lighting	+6.8 dB	+0.0567	White balance handling	90%
	Low-light scenarios	+5.6 dB	+0.0445	Noise minimization	79%
Multi-text Density	High-density (3+ instances)	+5.9 dB	+0.0489	Inter-text artifact reduction	77%
	Overlapping regions	+6.4 dB	+0.0534	Boundary handling	85%
	Multi-language mixed	+5.2 dB	+0.0423	Cross-linguistic consistency	73%
	Variable-sized clusters	+6.1 dB	+0.0501	Adaptive attention allocation	81%

**Table 13:** Comprehensive comparison of TSLEPS with existing methods.

Method	Detection Accuracy	PSNR	FLOPs	Mobile FPS
YOLOv9 + EraseNet	94.1%	27.16	57.84 G	3.42
EnsNet	N/A	27.22	10.53 G	0.32
MTRNet++	N/A	31.56	196.74 G	0.06
FETNet	N/A	30.38	55.05 G	2.13
TSLEPS (Ours)	97.5%	38.21	56.15 G	5.63

**Table 14:** Performance across different languages and imaging conditions.

Text Category	Detection Accuracy	Recall	PSNR	SSIM
Latin Script (High-res)	97.5%	96.4%	38.21	0.9607
Chinese Characters	95.2%	94.1%	36.43	0.9412
Arabic Script	91.8%	89.7%	33.76	0.9105
Handwritten Text	89.4%	87.3%	32.81	0.8957
Low-light Capture	88.7%	85.9%	31.62	0.8834

## Data Availability

The data presented in this study are available on request from the corresponding author. The data are not publicly available due to privacy.
